# Epidermal Notch1 recruits RORγ^+^ group 3 innate lymphoid cells to orchestrate normal skin repair

**DOI:** 10.1038/ncomms11394

**Published:** 2016-04-21

**Authors:** Zhi Li, Tom Hodgkinson, Elizabeth J. Gothard, Soulmaz Boroumand, Rebecca Lamb, Ian Cummins, Priyanka Narang, Amy Sawtell, Jenny Coles, German Leonov, Andrea Reboldi, Christopher D. Buckley, Tom Cupedo, Christian Siebel, Ardeshir Bayat, Mark C. Coles, Carrie A. Ambler

**Affiliations:** 1 School of Biological and Biomedical Sciences, Biophysical Sciences Institute, Durham University, Durham DH1 3LE, UK; 2 Centre for Immunology and Infection, Department of Biology and Hull York Medical School, York YO10 5DD, UK; 3 Institute for Inflammation and Repair, University of Manchester, Manchester M1 7DN, UK; 4 Department of Microbiology and Immunology, University of California, San Francisco, California 94143, USA; 5 MRC Centre for Immune Regulation, University of Birmingham, Birmingham B15 2TT, UK; 6 Department of Hematology, Erasmus University Medical Center, Rotterdam 3015CN, Netherlands; 7 Department of Molecular Biology, Division of Research, Genentech Inc, South San Francisco, California 94080, USA

## Abstract

Notch has a well-defined role in controlling cell fate decisions in the embryo and the adult epidermis and immune systems, yet emerging evidence suggests Notch also directs non-cell-autonomous signalling in adult tissues. Here, we show that Notch1 works as a damage response signal. Epidermal Notch induces recruitment of immune cell subsets including RORγ^+^ ILC3s into wounded dermis; RORγ^+^ ILC3s are potent sources of IL17F in wounds and control immunological and epidermal cell responses. Mice deficient for RORγ^+^ ILC3s heal wounds poorly resulting from delayed epidermal proliferation and macrophage recruitment in a CCL3-dependent process. Notch1 upregulates TNFα and the ILC3 recruitment chemokines CCL20 and CXCL13. TNFα, as a Notch1 effector, directs ILC3 localization and rates of wound healing. Altogether these findings suggest that Notch is a key stress/injury signal in skin epithelium driving innate immune cell recruitment and normal skin tissue repair.

## Abstract (web summary)

In normal skin, Notch directs keratinocytes to terminally differentiate. Here the authors show that Notch1 has a wider role in skin repair; Notch1 is activated in keratinocytes after damage and drives transcription of TNFα and inflammatory chemokines, which in turn recruit ILC3s and macrophages that promote repair.

## Graphical abstract

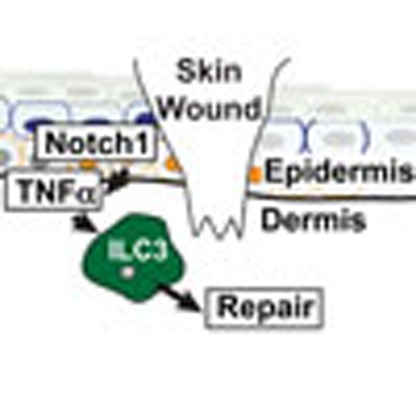

Wound healing in epithelial tissues involves three phases; inflammation, proliferation and remodelling^1^,^2^. Following injury, neutrophils and phagocytic cells infiltrate clearing microbial contaminants. The subsequent proliferative phase is characterized by epidermal proliferation, extracellular matrix deposition, granulation, vascularization and wound contraction. Following wound closure, excess cells and debris are removed in the remodelling phase and the extracellular matrix is reorganized to restore tissue strength. At the wound site, adaptive and innate immune cells regulate the skin wound healing process through production of cytokines, antimicrobial peptides and growth factors. Wound healing is not obligate on a functional immune system; most immune-deficient models heal wounds^3^,^4^. However, research shows macrophages have key roles in wound closure^5^. Further in mice, injury activates epidermal-resident γδ T cells called dendritic epidermal T cells (DETCs) to produce growth factors and inflammatory cytokines that control the epithelial injury response^6^. Whether the immune system helps or hinders effective repair remains controversial. Athymic nude mice undergo complete, scarless repair^3^,^7^, while conversely, macrophage persistence in wound sites leads to fibrosis and scar formation; thus proper timing of immune cell entry and exit is critical for normal repair^1^,^8^.

The Notch pathway is a key, cell-autonomous signalling pathway that directs cell fate and has pleiotropic functions in the skin^9^,^10^,^11^. Notch signalling initiates when a Notch ligand binds to one of the four receptors present on mammalian cells, which causes receptor cleavage and enables the intracellular domain to undergo nuclear translocation, and effect changes in gene transcription^12^. Expression of all four Notch receptors in the epidermis has been reported^13^. However, genetic studies suggest Notch1 and Notch2 are the primary receptors needed to regulate the cell differentiation required to maintain hair and skin epithelia^14^. A previous study using topically-applied pan-Notch activators and inhibitors suggested that Notch might be involved in wound healing^15^, and our previous work showed that forced, ectopic epidermal Notch1 activity resulted in extensive epidermal proliferation and severe inflammation, two phenotypic hallmarks of skin wound healing^9^,^16^,^17^.

Innate lymphoid cells (ILCs) are rare populations of lymphocytes that have key roles in secondary lymphoid tissue formation, homoeostasis and rapid production of cytokines in response to pathogen infection. ILCs are classified by cytokine production and expression of transcription factors into three groups, termed group 1, 2 or 3, and are found largely within the stroma of mucosal tissues^18^. Group 1 or ILC1s in mucosal epithelium produce interferon-γ-mediated responses against pathogens and are thought to contribute to intestinal pathologies when dysregulated, while Group 2 (ILC2s) are linked to allergenic responses and, in skin, to atopic dermatitis through the production of type 2 cytokines, IL5 and IL13 (refs 19, 20, 21, 22, 23). Group 3 or ILC3s are characterized by the expression of RORγ transcription factor and have key roles in the maintenance and repair of epithelial tissues. Further, ILC3s contribute to intestinal epithelial repair through IL22 upregulation^24^, and through ILC3-mediated release of granulocyte macrophage colony stimulating factor (GM-CSF) controlling macrophage and dendritic cell responses to gut commensal microflora^25^,^26^. In skin, recent studies have linked ILC3s to the pathogenesis of psoriasis through IL23a stimulated IL17 and IL22 production. However, despite the similarity of skin and intestine as barrier organs, the contribution of ILCs to the physiology of normal skin tissue repair remained unstudied.

In this study, we show that damage activates epidermal Notch signalling. Notch in turn, controls dermal entry of inflammatory cell subsets, including NKp46^low/–^CD4^+^ILC3s to wound sites. In uninjured mouse and human skin, dermis-resident ILC3s are exceptionally rare, but Notch-controlled epidermal signals recruit ILC3s in a TNFα-dependent process. Furthermore, using RORγ^+^-deficient mice, we present evidence that RORγ^+^ ILC3s, in wounds, produce IL17F and CCL3 (also known as MIP1α) and have key roles the normal healing response; ILC3s control epidermal proliferation and macrophage entry into the dermis.

## Results

### Skin injury activates Notch1 signalling

**Figure 1:**
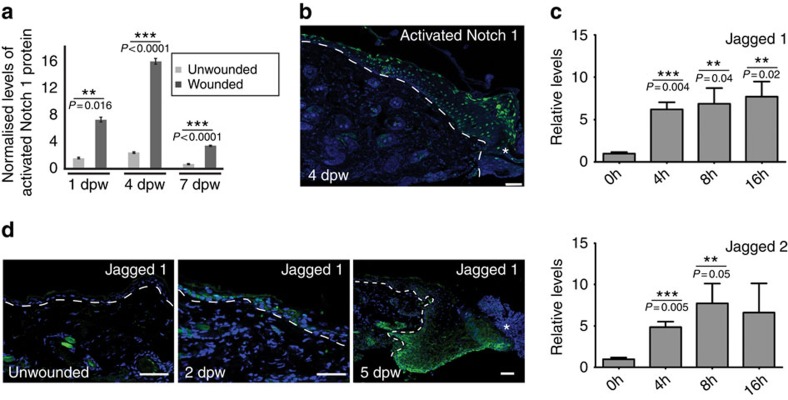
Notch1 is activated in wounded skin; the Notch ligands Jagged1 and Jagged2 are upregulated in response to injury. Back skin tissues from punch-wounded wildtype mice were collected 4, 8, 16 h and 1, 2, 4, 5 and 7 dpw. (**a**) Graph summary of cleaved, activated Notch1 protein levels quantified by antibody detection in a western immunoblotting assay (Supplementary Fig. 9a). Notch1 levels were standardized to β-actin levels as a loading control and normalized to Notch1 levels (designated as 1) in uninjured littermate mice. Unwounded back skin was taken from a punch-wounded mouse at a distal site (minimum 2 cm) from wound site. *n*=3 biological replicates each time point post wounding. Normally-distributed data was compared by Student's *t*-test between wounded and unwounded samples. (**b**) Section stained with antibodies specific for activated Notch1 on tissue collected 4 dpw. Note activated Notch1 is detectable in all epidermal layers. (**c**) mRNA levels of Jagged1 or Jagged2 quantified by qPCR in unwounded (0 h) and wounded back skin 4, 8, 16 h (h) post wounding. mRNA levels were normalized to UW. (**d**) Sections were stained for anti-Jagged 1 (green) on unwounded, 2 and 5 dpw. White asterisks (**b**,**d**) mark wound site; white dash line denotes epidermal/dermal boundary (**b**,**d**). Graph bars represent experimental mean of 3 or more biological replicates and error bars represent standard error of the mean (s.e.m.); Results statistically significant marked with ** or ***; *P*-values included on graphs. Scale bars equal 50 microns. All experiments were repeated ⩾2 times.

To determine the roles of Notch in wound healing, 6-week-old mice were given two, full-thickness punch wounds, and back skin tissues were analysed for Notch1 at 1, 3/4 and 7 days post wounding (dpw). Wounding caused an immediate increase (∼7 × ) in Notch1 activity 1 dpw with peak activity detected 4 dpw (∼16 × ) (Fig. 1a). Stained sectioned tissues revealed Notch1 was primarily active in suprabasal epidermal keratinocytes 1 and 4 dpw (Fig. 1b and Supplementary Fig. 1). A significant increase in Jagged1 and Jagged2 messenger RNA (mRNA) expression was detected by quantitative real time PCR (QPCR) within 8 h of injury (Fig. 1c). Published studies show skin dendritic cells in the mouse ear can upregulate the Notch ligands Jagged1 and Jagged2 in response to damage^27^, however we detected Jagged1 protein in basal and suprabasal epidermal layers 2 and 5 dpw. This suggests that Notch ligands are available locally in the epidermis to activate Notch during wound healing (Fig. 1d). We conclude that the Notch pathway is active in the inflammatory and proliferation phases of wound healing.

### Epidermal Notch1 drives dermal immune responses after injury

**Figure 2:**
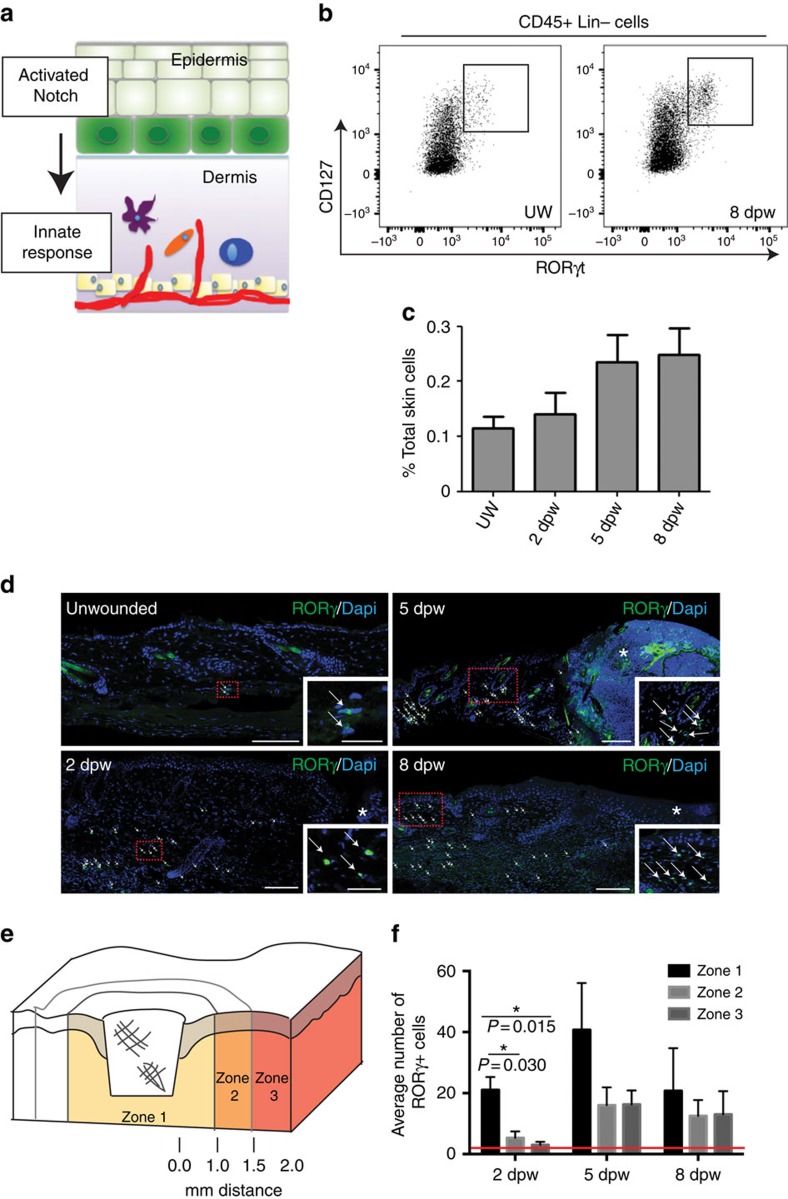
ILC3s populate dermis in response to skin wounding. (**a**) Schematic of experimental hypothesis; Notch activation following injury in keratinocytes (green) induces innate immune cell recruitment (represented by purple, blue and orange cells) into the underlying dermis. (**b**,**c**) Dorsal back skin from UW or punch-wounded (4 mm biopsy) wildtype animals were collected 2, 5 or 8 dpw. Whole back skin tissues were digested to single cell suspensions and co-stained with a lineage antibody panel (Lin) that react with cells from the major hematopoietic cell lineages and antibodies to CD45, CD127 and RORγt. (**b**) Cells were first gated for CD45+ and Lin–, then analysed for co-expression of CD127 and RORγt. (**c**) The percentage of CD45^+^Lin^−^CD127^+^RORγt^+^ cells (ILC3s) in the total cell population was calculated for UW or wounds 2, 5 and 8 dpw. Per cent (%) total skin cells; averages of three biological replicates. (**d**) Back skin sections from unwounded mice or from punch-wounded (4 mm) tissues collected 2, 5 or 8 dpw stained with an antibody to RORγ (green). Embedded insets are higher magnification images of the boxed region in the accompanying panel. RORγ^+^ cells are marked by white arrows and wound site marked by white asterisk. (**e**,**f**) Quantification of RORγ^+^ cells adjacent to wounds analysed by two-way ANOVA with statistical significance (marked with a single black asterisk, **f**) between zones calculated by Bonferroni's multiple comparison test. Zone 1=area from edge of wound to 1 mm distal from wound edge, zone 2=region 1–1.5 mm distal from wound edge and zone 3=region 1.5–2 mm distal from wound edge. (**f**) Red line denotes average number of RORγ^+^ cells in unwounded dermis (1.47±0.87 per mm^2^). Graph bars are average cell numbers from 3 sections from 3 biological replicates; scale bars equal 100 microns.

Our previous work showed that epidermal Notch activity, via Jagged1, stimulates epidermal production of pro-inflammatory TNFα and persistent Notch activity leads to CD3^+^CD4^+^ T cell recruitment^9^,^17^. Thus we hypothesized that Notch signalling leads to inflammatory cell recruitment following injury (Fig. 2a). We quantified populations of dermal immune cells in unwounded skin (UW) and punch-wounded skin during the inflammatory, proliferation and early remodelling phases at 2, 5 and 8 dpw, respectively. Wounding induced the expected early influx of CD11b^+^Gr-1^+^ polymorphonuclear neutrophils and CD11b^+^F4/80^+^ monocytes/macrophages (Supplementary Fig. 2a,b). The percentage of anti-inflammatory M2 macrophages was determined by CD206 marker expression; in alignment with previously published studies, the macrophage population was heterogeneous throughout skin repair, with 78–87% of macrophages expressing the M2 marker at all wound stages investigated (Supplementary Fig. 2d)^28^. CD206 marker expression was quantified on spleen cells as an experimental control and, in agreement with published studies, we detected ∼20% of splenocytes expressing CD206 (Supplementary Fig. 2d). NK1.1^+^ (NK and/or NKT) cell numbers peaked at 8 dpw (Supplementary Fig. 2c) consistent with a role in tissue remodelling. Next, we examined the innate lymphoid cell populations, as these cells were known to have roles in intestinal tissue repair and skin pathology. Increased numbers of ILC2s were detected by 8 dpw; while an increase in ILC1s was detected at 5 and 8 dpw during the late proliferation and remodelling phases of wound healing when IFNγ levels are high (Supplementary Fig. 2e,f)^19^. RORγ^+^CD127^+^Lin^–^ ILC3s were detected, yet these cells comprised a very small proportion of total back skin cells, <1% of total dermal cells, at all time points (Fig. 2b,c). In summary, our data show that injury controls localized and temporal recruitment of leukocytes including ILC subsets.

We theorized that the small numbers of ILC3s detected by flow cytometry were likely due to the inclusion of dermal cells from both wounded and non-wounded skin. To test if wounding caused localized recruitment of ILC3s to wound sites, we examined the distribution of RORγ^+^ ILC3s in sectioned wound tissues (Fig. 2d). At all time points post wounding ILC3s were detected in the dermis. At 2 dpw, most ILC3s were found in the reticular dermis, however by 5 and 8 dpw ILC3s were detected in both the reticular and papillary dermis, suggesting active recruitment of cells from the reticular blood vascular network into the dermis (Fig. 2d). Similarly in human skin, we detected infiltration of dermal RORγ^+^CD127^+^CD3^–^ ILCs following wounding using a punch biopsy (Supplementary Fig. 3a). In mice tissues, the distribution of ILC3s was quantified relative to the epidermal wound edge as defined by the distinctive phenotypic appearance of this tissue (Fig. 2e,f). Most RORγ^+^ ILC3s were localized nearest to the wound site within 1 mm of the wound edge (zone 1) and maximal numbers were detected at 5 dpw during the proliferative phase (Fig. 2d). As controls, sections from wounded RORγ^−/−^ mice were stained with RORγ antibodies; no staining was detected confirming specific immunoreactivity of RORγ antibodies in tissue sections (Supplementary Fig. 4a). ILC3 subsets differentially express NKp46 and CD4, so we analysed the surface marker expression in RORγ^e*GFP*/*+*^ skin (Supplementary Fig. 4b)^29^. The majority of RORγ-eGFP^+^ cells were CD4^+^, and expressed very low levels of NKp46 with heterogeneous expression of CD117, thus CD4^+^NKp46^low/–^ ILC3s. Further, these ILC3s expressed the chemokine receptors, CCR6, CXCR4 and CXCR5, important for ILC3 chemotaxis (Supplementary Fig. 4b).

**Figure 3:**
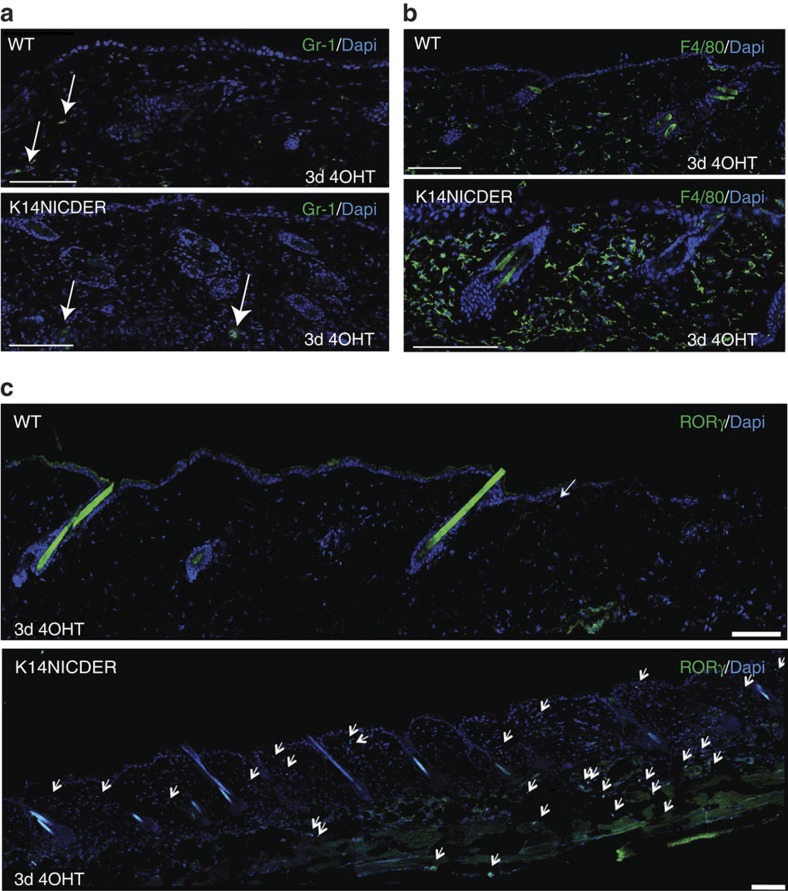
Epidermal Notch activation drives dermal recruitment of ILC3s and F4/80^+^ macrophages. Back skin sections of K14NICDER transgenic and littermate control mice treated with 4OHT for 3 days and stained with antibodies to Gr-1 (**a**, green), F4/80 (**b**, green) or RORγ (**c**, green). White arrows mark either Gr-1^+^ (**a**) or RORγ^+^ (**c**) cells. Note rare Gr-1^+^ neutrophils (**a**, white arrows) were detected in transgenic and control mice, however F4/80^+^ (**b**) and RORγ^+^ (**c**, white arrows) cells are abundant in stained K14NICDER back skin sections; scale bars equal 100 microns; experiments were repeated ⩾3 times.

Having established the timed cascade of immune cell infiltration in skin wounds, we next investigated the link between injury-activated epidermal Notch and the dermal accumulation of ILC3s and other immune cell subsets. Previously we published a K14NICDER transgenic mouse model where Notch1 activity can be tightly controlled in the epidermis by drug application. A full characterization of this mouse model has been previously published^9^,^17^. In brief, the K14NICDER transgene contains a truncated, Notch1 intracellular domain (NICD) that can be temporally and spatially activated by 4-hydroxy-tamoxifen (4OHT) in the basal, keratin 14-expressing epidermis. Here, Notch was activated by 4OHT in transgenic mice for 3 days. Control mice were likewise 4OHT treated and sectioned tissues were antibody stained to visualize Gr-1^+^ neutrophils, F4/80^+^ macrophages and RORγ^+^ ILC3s (Fig. 3a–c). Only rare neutrophils were detected in both 4OHT-treated transgenics and controls (Fig. 3a), but a substantial increase in the number of macrophages and ILC3s were detected in transgenic dermis compared to control tissue (Fig. 3b,c), suggesting that epidermal Notch activation in the absence of tissue damage is sufficient to drive recruitment of subsets of wound-induced dermal immune cells.

**Figure 4:**
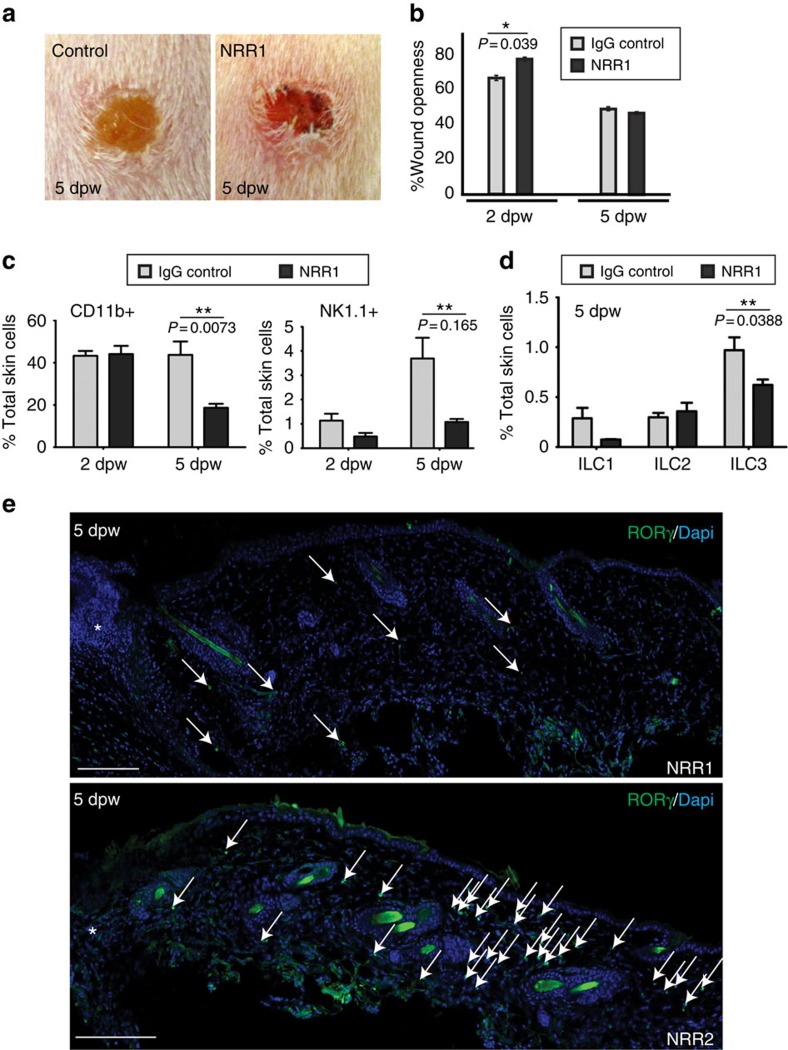
NRR1 inhibition of Notch1 dampens the immune cell response after injury. Punch-wounded wildtype mice injected i.p. with 5 mg kg^−1^ NRR1 (**a**–**e**), NRR2 (**e**) or IgG isotype control (**a**–**d**) antibodies for 7 days before injury. (**b**) Wound openness was measured as a per cent of initial wound size at 2 and 5 dpw. NRR1-treated wounds were significantly more open 2 dpw (*P*=0.039; *) but not 5 dpw. (**c**–**e**) Dorsal back skin from punch-wounded NRR1 or control IgG treated mice was collected 2 or 5 dpw. Single cell suspensions of whole back skin were co-stained with antibodies against Lineage markers (Lin), CD45, CD3, CD11b, CD25, ST2, NK1.1, CD127, F4/80 and/or RORγt prior to analysis by flow cytometry. Cells were initially gated on the basis of CD45 expression and immune cell numbers quantified on the basis of expression of diagnostic surface markers: CD11b (polymorphonuclear neutrophils/monocytes/macrophages), NK1.1 (NK/NKT cells), F4/80^−^CD3^−^CD127^+^NK1.1^–^ (ILC1s), Lin^−^CD127^+^CD25^+^ST2^+^ (ILC2s), Lin^−^CD127^+^ RORγt^+^ (ILC3s). Graphs represent the average per cent (%) of total skin cells from three biological replicates. Student's *t*-test (**b**–**d**) between NRR1-treated and control-treated samples was used to determine significance marked with **; *P*-values included on graphs. (**e**) Sections of wounds 5 dpw from NRR1 or NRR2-treated mice stained with an antibody to RORγ (green). RORγ^+^ cells marked by white arrows and wound sites marked with white asterisks. Wounding recruits RORγ^+^ cells into the dermis; pre-treatment with NRR1, but not NRR2 receptor blocking antibodies inhibits RORγ^+^ cell infiltrate after wounding; scale bars equal 100 microns.

Next, we inhibited Notch1 receptor activation using NRR1 blocking antibodies, which block ligand binding to Notch1 receptor but not the other Notch receptors, thus specifically inhibiting Notch1 signalling^30^. As controls, mice were treated with NRR2 blocking antibodies, which have equivalent efficacy against Notch2 receptors, isotype IgG antibodies or phosphate buffered saline (PBS). Mice were injected intraperitoneally (i.p.) for 7 days before wounding, and we confirmed that antibody treatment caused expected systemic effects; NRR1-treated mice were blocked in thymocyte development and NRR2-treated mice were blocked in marginal zone B cell maturation, Notch1 and Notch2 dependent processes, respectively (Supplementary Fig. 5a)^30^. Following wounding, closure rates were measured and tissues collected 2 or 5 dpw. Wounds treated with the Notch1 blocking antibody, NRR1, were significantly more open (77%) 2 dpw compared to mice injected isotype IgG control (66%), but by 5 dpw wound size in NRR1-treated mice did not differ from controls (Fig. 4a,b). NRR1-treated wounds displayed a distinct phenotype; wounds showed increased erythema, with domed margins (signs of abnormal wound granulation), compared to controls (Fig. 4a). Following treatment with NRR1, detectable nuclear activated Notch1 was reduced in the epidermal layers as detected by antibody staining, and total cleaved, active Notch1 protein was reduced in whole skin preparations quantified by western immunoblotting (Supplementary Fig. 5c,d). No difference in basal and suprabasal marker expression was detected in NRR1 and control-treated skin suggesting NRR1 treatment did not block differentiation of epidermal keratinocytes (Supplementary Fig. 5e).

The impact of reduced Notch1 activation on wound-recruited dermal immune cells was assessed in sectioned tissues and by flow cytometry in NRR1-treated mice. At 2 dpw, most dermal immune cell populations were similar in NRR1 and IgG control-treated wounds (Fig. 4c; Supplementary Fig. 5f) with the exception of ILC3s; fewer dermal ILC3s were detected adjacent to wounds at 2 dpw (Supplementary Fig. 4f). At 5 dpw, reduced Notch1 levels led to fewer dermal CD11b^+^ leukocytes, NK1.1^+^ cells, ILC1s and ILC3s (Fig. 4c,d). We confirmed that Notch1, but not Notch2 activity was directly linked to RORγ^+^ ILC3 recruitment; ILC3 recruitment was inhibited in wounded mice treated with NRR1, but not NRR2, blocking antibodies (Fig. 4e).

Taken together these results demonstrate that Notch, as a damage response factor, directs recruitment of dermal immune cells. Further, we found a novel link between epidermal Notch activity and ILC3 recruitment to wound sites. Given the key roles of ILC3s in regulating mucosal tissue repair, we next investigated the Notch-regulated mechanism leading to ILC3 recruitment and their functions in skin repair.

### Epidermal Notch1 drives TNFα-mediated recruitment of ILC3s

**Figure 5:**
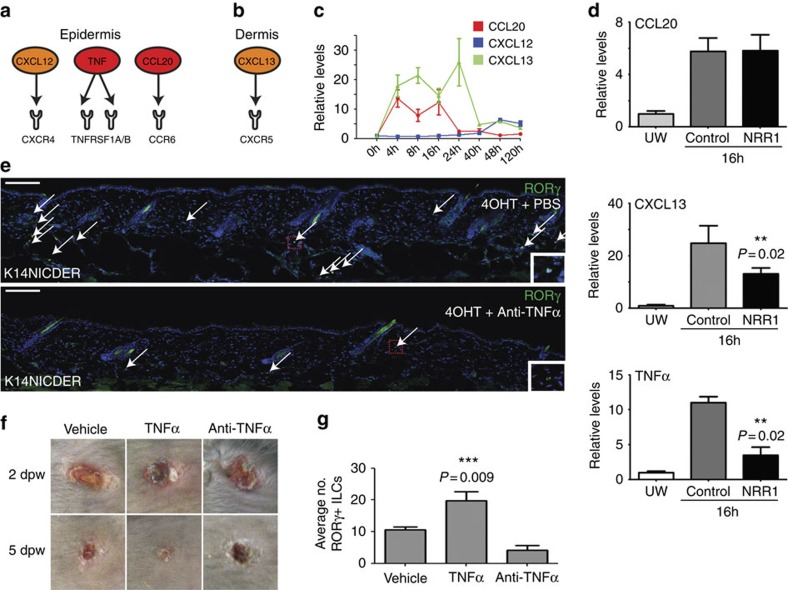
Notch1 drives expression of TNFα that regulates ILC3 localization in wounds. (**a**,**b**) Factors upregulated by Notch in the (**a**) epidermis and (**b**) dermis that can interact with receptors expressed on RORγ^+^ ILCs. (**c**) mRNA levels of CCL20, CXCL12 and CXCL13 quantified by qPCR in unwounded and wounded back skin 4, 8, 16, 24, 40, 48 and 120 hrs post wounding. (**d**) qPCR quantitation of CCL20, CXCL13 and TNFα mRNA in unwounded or wounded mice injected with NRR1 blocking antibodies or control IgG antibodies for 7 days before wounding and analysed 16 hrs after wounding. Graph bars represent mean of biological replicates (*n*=3). (**e**) Back skin sections of K14NICDER transgenic mice injected i.p. with PBS or TNFα blocking antibody for 7 days followed by 2 days of 4OHT-treatment labelled with antibodies to RORγ (green) and DAPI counterstained. Embedded insets are higher magnification images of the boxed region in the accompanying panel. (**f**) Punch wounds treated with PBS (vehicle), TNFα or TNFα blocking antibody (anti-TNFα) photographed at 2 and 5 dpw. Note red, inflamed tissues evident adjacent to TNFα-treated wounds 2 dpw. Experiment repeated twice (total animal numbers *n*=4 control; *n*=7 TNFα blocking antibody; *n*=8 TNFα). (**g**) Quantification of dermal RORγ^+^ cells in wounded mice 2 dpw treated with vehicle, TNFα or TNFα blocking antibody (*n*=3 control; *n*=5 TNFα; *n*=5 TNFα antagonist; sample number=biological replicates; quantified area <1,600 micrometres distal to wound site). All panels: white arrows mark RORγ^+^ cells. Error bars on graphs represent standard error of the mean (s.e.m.). Scale bars equal 100 microns. Results statistically significant as determined by Student's *t*-test (**d**,**g**) marked with ** or ***; *P*-values included on graphs.

To investigate the mechanism by which Notch1 leads to ILC3 recruitment, dermal and epidermal gene expression profiles of uninjured, K14NICDER transgenic mouse back skin (NIH GEO:GSE23782 (ref. 9)) were examined. IPA software was used to identify secreted factors upregulated in 4OHT-treated K14NICDER skin (6 × or greater) that could facilitate immune cell infiltration into the dermis and the subsequent activation (Fig. 5a,b). Analysis identified several key candidates that could regulate innate immune cell recruitment and activation (NIH GEO: GSE29777 (ref. 31)). Of these, Notch-regulated factors CCL20 (14.1 × ), CXCL12 (8.0 × ) and CXCL13 (6.5 × ) were of particular interest as these factors directly regulate ILC3s; and we showed that the cognate receptors CCR6, CXCR4 and CXCR5 for the ligands CCL20, CXCL12 and CXCL13, respectively, are expressed on wound-recruited ILC3s (Supplementary Fig. 4b and Fig. 5a,b)^32^. Further, TNFα (17.1 × ) was also of interest, as it can direct CCL20 expression in the epidermis^33^ and CCL20 is a key recruitment factor for RORγ^+^ ILC3s in lymphoid tissues^34^.

The ligand/receptor pairings of CCL20/CCR6, CXCL12/CXCR4 and CXCL13/CXCR5 have vital chemotactic functions in many immune cells including ILCs, dendritic cells (DCs) and T and B cells. Innate immune cell recruitment in mucosal epithelium is CCL20/CCR6 dependent^35^. Skin wounding caused robust, early activation of Notch1 (Fig. 1b), thus we measured CCL20, CXCL12 and CXCL13 mRNA levels at early time points (4, 8, 16, 24, 40, 48 and 120 h) after injury by quantitative PCR (qPCR) (Fig. 5c). CCL20 and CXCL13 levels were elevated immediately following wounding and returned to near background levels by 40 h post injury and remained low (Fig. 5c). No increase in CXCL12 mRNA levels was detected from 0 to 24 h post wounding, with a moderate increase in CXCL12 levels after 48 h. In human wounds, CXCL13 and CCL20 were also differentially regulated with highest expression observed early during the inflammatory phase (Supplementary Fig. 3b). The early activation of CCL20 and CXCL13 in wounded tissues, and in Notch-active transgenic mice, suggests that Notch has a role in their activity. Mice with reduced Notch activity (NRR1 treated) had lower levels of CXCL13, but not CCL20 compared to controls (Fig. 5d). These data link Notch activity to CXCL13 activation, yet we cannot rule out a role for Notch1 in CCL20 activation as in our model Notch activity is reduced, not fully blocked. However, CXCL13 and CCL20 were transcriptionally downregulated within 24-h of injury, while Notch1 activity remained high (Fig. 1a) suggesting other transcriptional regulators are also likely important.

To test whether CCL20 and CXCL13 are essential chemokines in ILC3 recruitment into skin wounds, we examined ILC3 recruitment in mutant mice deleted for single chemotactic pathways. CCR6^−/−^ and CXCL13^−/−^ mice and their respective littermate controls were punch wounded; wound closure rates were monitored and tissue sections were analysed by immuno-detection for RORγ^+^ ILC3 recruitment at 5 dpw. There was no detectable difference in wound closure rates between the mutant and control mice. The number of dermal ILC3s in antibody-stained CXCL13^−/−^ mutant tissue sections was not significantly different from controls (Supplementary Fig. 6a,b), whereas, a small increase in ILC3 numbers was detected in CCR6^−/−^ mutants compared to controls (Supplementary Fig. 6c,d). We identified injury-induced upregulation of a third chemokine in human wounds, CXCL16, that is known to regulate the chemotaxis of both mouse ILC3s and human innate immune cells (Supplementary Fig. 3b)^29^,^36^. Taken together these results suggest multiple, redundant pathways are important in ILC3 chemotaxis.

Lastly, we found levels of TNFα corresponded to levels of Notch activity in skin with increased (K14NICDER) and decreased Notch activity following injury (wounded NRR1-treated mice) (Fig. 5a,d)^9^. Thus, we determined if TNFα downstream of epidermal Notch activity mediates ILC3 localization. In unwounded K14NICDER, TNFα activity was blocked by injecting a TNFα blocking antibody, or PBS control, daily for 7 days before 4OHT-activation of Notch. Antibody staining on tissue sections revealed that anti-TNFα treatment blocked Notch-mediated ILC3 localization into the skin (Fig. 5e).

To confirm that TNFα regulates ILC3 dermal localization in wounds, wounded animals were treated with recombinant TNFα, TNFα blocking antibody or PBS daily. By 5 2dpw, a substantial difference was observed between the three groups of mice: TNFα accelerated wound closure and increased ILC3 numbers (1.8 × ), while the antagonist inhibited closure with reduced numbers of ILC3s (0.4 × ) (Fig. 5f,g and Supplementary Fig. 6e). Mechanistically, TNFα bioavailability may impact ILC3 recruitment through regulating CCL20. Previous studies have shown that CCL20 is produced from epidermal keratinocytes in response to TNFα stimulation^33^, and we confirmed that primary dermal cells respond similarly (Supplementary Fig. 6f). However, it is almost certain that multiple factors control CCL20 expression; TNFα alone or in combination with IL-1β, CD40 ligand, IFNγ, and IL17 can all stimulate CCL20 production in epidermal keratinocytes and dermal fibroblasts (Supplementary Fig. 6f). In summary, these results implicate a Notch/TNFα mechanism, working in conjunction with chemokines, in ILC3 recruitment into skin wounds.

### The functional role of Notch-recruited ILC3s in skin wounds

**Figure 6:**
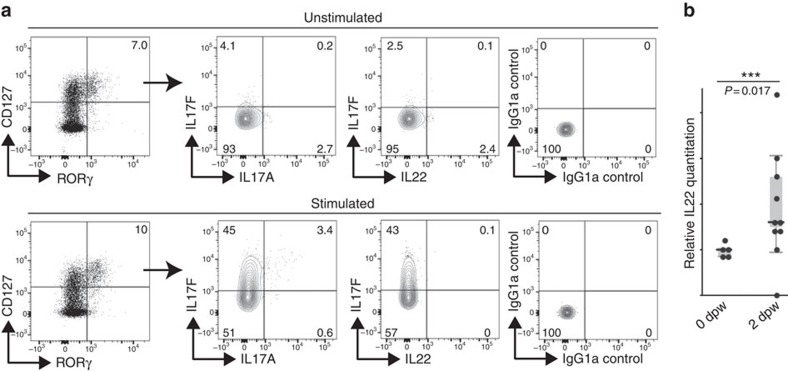
Wound-recruited ILC3s produce IL17F. (**a**) Cytokine production profile of CD45^+^Lin^−^CD127^+^RORγ^+^ILC3s assessed by flow cytometry with intracellular staining. Total skin cells unstimulated or stimulated for 3 h in the presence of PMA and ionomycin were analysed. Numbers indicate the percentage of cells in respective quadrants. Analysed cells pooled from eight biological replicates. IgG1a isotype antibodies were included as controls for the intracellular staining. (**b**) mRNA was isolated from unwounded (0 dpw, *n*=5) or wounded RORγ^+/−^ (*n*=6) skin collected 2 dpw. Relative mRNA levels of IL22 were quantified by qRT-PCR. mRNA levels were normalized to unwounded controls (designated 1). Data plotted using a quartile, box and whisker plot. Data were not normally distributed and the medians of experimental replicates were analysed by Mann–Whitney test. Wounding induced statistically significant upregulation of IL22 (***, *P*=0.0170).

Innate lymphocytes and other cell types, including Th17 cells, DETCs and dermal γδ T cells, can be potent sources of IL17 and IL22 cytokines that attenuate immune responses^18^,^38^,^39^,^40^. IL17A production drives psoriatic inflammation in skin^41^ and imiquimod-induced psoriatic-like lesions in mice express high levels of IL17A and IL17F^42^. Further, the IL22 receptor signalling pathway has a key role in skin wound healing^43^ and IL22 has a key role in thymus regeneration^20^ and accelerates keratinocyte closure in *in vitro* wound scratch assays^24^. ILC3s isolated from 5 dpw dermis were analysed for cytokine production by intracellular flow cytometry analysis (Fig. 6a). We analysed IL17A, IL17F and IL22 cytokine production in unstimulated and phorbol 12-myristate 13-acetate (PMA)/ionomycin-induced cells. Although, we detected low levels of IL22-, IL17A- and IL17F-producing cells among the population of unstimulated ILC3s, following PMA/ionomycin, the majority of ILC3s predominantly expressed IL17F, with a small fraction of cells double positive for IL17A and IL17F. Similarly, human wounds populated with dermal ILC3s have elevated levels of IL17F and IL17A (Supplementary Fig. 3c). IL22 is difficult to detect by flow cytometry, so in addition we examined mRNA levels by qRT-PCR. We detected increased IL22 levels by 2 dpw consistent with a potential role for ILC3s in producing IL22 needed for skin repair (Fig. 6b). In summary, results suggest that ILC3s contribute to normal physiological tissue repair in the skin through production of IL17F and to a lesser extent IL17A and IL22.

**Figure 7:**
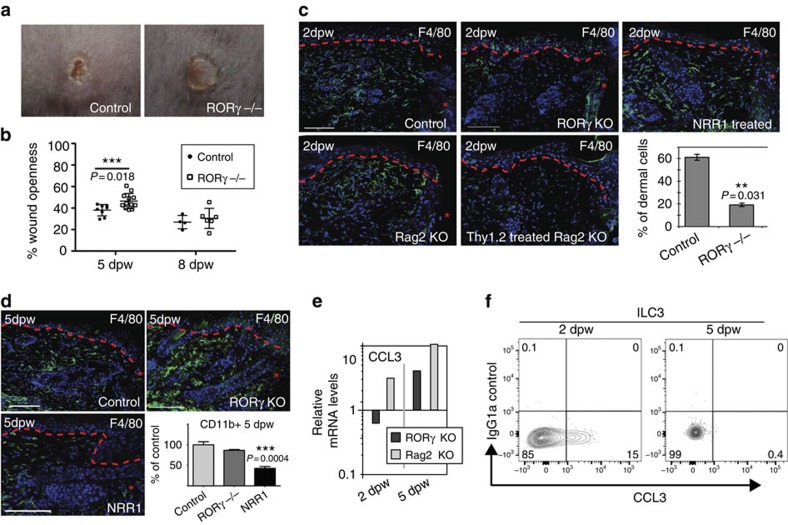
Notch-recruited ILC3s orchestrate macrophage entry and wound closure through production of CCL3. (**a**,**b**) 4 mm punch-wounded RORγ^+/−^ (control) and RORγ^−/−^. (**b**) Quantitative analysis using 3D imaging; wound openness is measured in control and RORγ^−/−^ wounds at 5 dpw and 8 dpw. The mean openness of RORγ^−/−^ wounds is significantly greater than that of wildtype mice at 5 dpw (*n*=12 RORγ^−/−^; *n*=8 control) but not 8 dpw (*n*=6 RORγ^−/−^; *n*=4 control) (*F*=6.721; *df*=18; *P*=0.018). (**c**,**d**) Sections of wounded RORγ^−/−^ (RORγ KO), RORγ^+/−^/Rag2^−/−^ (Rag2 KO), RORγ^+/-^/Rag2^−/−^ injected with anti-Thy1.2 antibodies (Thy1.2 treated Rag2 KO), wildtype mice treated with NRR1 blocking antibodies (NRR1 treated) and/or wildtype control tissues collected 2 dpw (**c**) or 5 dpw (**d**) stained with antibodies to F4/80 (green) and DAPI counterstained (blue). Graph shows percentage of F4/80^+^ dermal cells quantified by cell counting on sections in control verses RORγ^−/−^mice 2 dpw. (**d**, bottom right panel). Flow cytometry analysis of single cell suspensions from parallel wounding experiments analysed 5 dpw. Cells stained with CD45 and CD11b prior to analysis (*n*=3 for each genotype/time point) and the relative number of CD45^+^CD11b^+^ cells was determined. Results were normalized (designated 100%) to controls from skin collected 5 dpw. (**e**) mRNA was isolated from unwounded controls (*n*=3) and punch-wounded RORγ^−/−^ (RORγ KO; *n*=3) or Rag2^−/−^ (Rag2 KO; *n*=3) skin collected 2 dpw or 5 dpw. Relative mRNA levels of CCL3 were quantified by qPCR. mRNA levels were normalized to wildtype skin 2 dpw (designated 1). Data plotted on a log scale; 3 biological replicates. (**f**) CCL3 production in PMA/ionomycin stimulated ILC3s quantitated by flow cytometry 2 and 5 dpw. Red, dashed line (**c**,**d**) marks epidermal–dermal boundary; single asterisks mark wound site; dpw=days post wounding; graph bars=mean and error bars=standard error of the mean (s.e.m.); scale bars=100 microns. Results statistically significant as determined by linear mixed effects model (**b**) or Student's *t*-test (**c**,**d**) marked with ** or ***; *P*-values included on graphs.

To investigate the function of Notch-recruited ILC3s in skin tissue repair, we examined the wound healing in RORγ^−/−^ and littermate controls (Fig. 7a). We developed non-invasive 3D imaging methods to quantify wound closure rates in these animals. Briefly, mice were photographed repeatedly using a 3D camera and an accurate measurement of the wound perimeter was calculated over time (Fig. 7b and Supplementary Fig. 7a–e). Wounds were significantly more open in RORγ^−/−^ mice given 4 mm punch wounds compared to control littermates at 5 dpw (Fig. 7a,b and Supplementary Fig. 6e). However, by 8 dpw, wounds were of a similar openness in mutant and control mice with re-epithelialization complete in both (Fig. 7b and Supplementary Fig. 7e). Wound-healing defects were exacerbated in RORγ^−/−^ mice with larger diameter (6mm) wounds. Larger wounds take longer to close and wound were significantly more open at 8dpw in RORγ^−/−^ mice receiving large wounds (Supplementary Fig. 7f). Delayed wound closure in RORγ^−/−^ mice with small wounds (4mm) was linked to delayed re-epithelialization of the keratinocyte layer; at 5 dpw, RORγ^−/−^ mice had a marked reduction in Ki67^+^ epidermal cells compared to controls (Supplementary Fig. 8a–c). We confirmed that phenotypes observed in the RORγ^−/−^ mice resulted from loss of RORγ^+^ ILC3s. We transplanted spleen cells from Rag2^−/−^ mice (lacking αβ and γδ T cells, but containing RORγ^+^ ILCs) into RORγ^−/−^ mice 24 h before wounding. Cell transplantation ameliorated wound pathology observed in control RORγ^−/−^ mice and donor RORγ^+^ ILCs could be detected adjacent to wound sites (Supplementary Fig. 8d). Therefore, loss of ILCs in RORγ^−/−^ mice directly causes wound pathology.

Crosstalk between ILC3s and macrophages has been shown to regulate macrophage function in intestinal homoeostasis^26^, thus we examined the inflammatory infiltrate in wounded RORγ^−/−^ mice. We detected no change in neutrophil, intra-epidermal macrophage and ILC2 numbers (Fig. 7c and Supplementary Fig. 8e,f), however, a threefold reduction in F4/80^+^ dermal macrophages was detected 2 dpw, as quantified in sectioned tissues (Fig. 7c, bottom right panel), compared to wildtype littermates. Rag2^−/−^ controls, which lack αβ and γδ T cells but contain ILC3s, had normal macrophage numbers. Anti-Thy1.2 antibodies were used to deplete ILCs in the Rag2^−/−^ background and 3.6-fold fewer ILC3s, and 2.7-fold decrease in ILC2s were detected by flow cytometry in antibody-treated animals 2 dpw (Supplementary Fig. 8g,h)^44^. Anti-Thy1.2-treated Rag2^−/−^ mice had deficient macrophage numbers at skin wound sites confirming that delayed macrophage recruitment resulted from specific loss of dermal ILC3s, not T lymphocytes, early in the wound-healing programme. In NRR1-treated mice where ILC3 numbers are reduced, but not to zero, F4/80^+^ macrophages could be detected, although few were found adjacent to the site of injury compared to controls (Fig. 7c).

By 5 dpw, F4/80^+^ cells were plentiful within the dermis in wildtype and RORγ^−/−^ wounds suggesting that loss of ILC3s causes a delay, but not a block in macrophage/monocyte infiltration (Fig. 7d). NRR1-treated animals had reduced numbers of F4/80^+^ macrophages at 5 dpw. Quantification by flow cytometry revealed similar trends; CD11b^+^ cell numbers were at similar levels in both mutant and control wounds by 5 dpw, while reduced in NRR1-treated animals (Fig. 7d, bottom right panel). These results are consistent with previous reports suggesting Notch1 has additional roles in regulating macrophage/monocyte responses to tissue damage^45^.

Decreased levels of CCL3, a key monocyte/macrophage recruitment chemokine, were detected in RORγ^−/−^ skin at 2 dpw compared to wildtype and Rag2^−/−^ controls, but not in day 5 wounds (Fig. 7e). We analysed whether ILC3s produce CCL3; CCL3 was detected in ILC3s isolated from 2 dpw, but not 5 dpw. This suggests that CCL3 production by ILC3s is required for early macrophage entry into wounded tissues in the healing programme (Fig. 7f).

## Discussion

**Figure 8:**
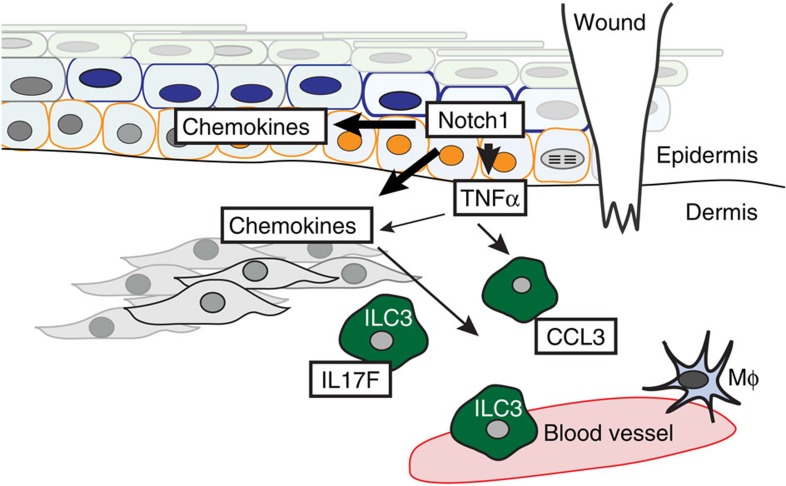
Notch1 orchestrates wound healing through control of type 3 innate lymphoid cells (ILC3s). Skin wounding activates Notch pathway (orange cells) in the basal and suprabasal epidermis. Epidermal Notch1 regulates chemokine production in epidermal and dermal cells; injury-induced Notch1 activity upregulates TNFα, which can induce CCL20 in epidermal and dermal cells (grey cells). TNFα downstream of Notch1 regulates ILC3 localization within the dermis surrounding the wound and ILC3s are key sources of IL17F and early CCL3 production in the wound-healing cascade. ILC3s subsequently influence epidermal proliferation and macrophage (Mφ) recruitment, two key events in the skin wound-healing programme.

Dysregulated Notch signalling has been linked to aberrant human and mouse wound healing and our previous work linked aberrant Notch activity with human keloid formation, however, the mechanistic detail or the site of action was unknown^9^,^15^,^46^,^47^,^48^. Here, using genetic and chemical tools to modulate Notch activity^17^,^30^,^49^, we have shown that Notch1 activity in epidermal keratinocytes has an essential temporal role in the wound-healing process through action on dermal immune cell recruitment and activation (Fig. 8). These findings are important, as it is clear that correct entry and exit of immune cells is essential for conventional repair. Notch1 levels were highest in the inflammatory and proliferation phases of the wound response, and during the later remodelling phase when the epithelium has closed^2^, Notch1 activity returns to levels detected in UW. Jagged1 and Jagged2 are similarly upregulated after injury and Jagged1 protein was detected in the epidermis adjacent to wounds, suggesting that Notch is activated through classical ligand–receptor interactions in wound repair. Reducing epidermal Notch1 activity inhibits wound closure and innate immune cell recruitment, and activating Notch1 in the epidermis without tissue or barrier damage is sufficient to drive expression of TNFα, CXCL13 and CCL20 in the skin and induce recruitment of ILC3s (Fig. 8).

Consistent with our findings using NRR1 blocking antibodies, wound healing is also impaired in Notch1^+/−^ mice; Notch1^+/−^ mice have reduced levels of TNFα and fewer F4/80^+^ macrophages near the site of injury^45^. The authors conclude these defects arise from loss of Notch1 activity in macrophages and inhibition of macrophage cytokine production^45^,^50^. However, our results suggest that reduced Notch signalling in epithelial keratinocytes in Notch1^+/−^ mice likely contributes to wound-healing defects. In our experiments, whole skin protein lysates were used and as such some cleaved Notch1 protein may come from macrophages, although the contribution is likely to be minimal. *In situ* antibody labelling showed strong nuclear expression of active Notch1 that was detected throughout the epidermis in the inflammatory phase. Moreover, at 7 dpw, when Notch1 levels decreased in wounds as detected by protein blotting, macrophages are still persistent within the dermis.

It is well established that Notch1 has a cell-autonomous role in lineage specification of cells within both adaptive and innate immune systems^51^. Here, we demonstrate that Notch activity in non-immune cells initiates an event cascade controlling inflammation and immune regulation in the skin. Neutrophil recruitment, however, was unaffected by changes in Notch1 activity (Fig. 2a). Neutrophil recruitment is mediated by the release of danger signals or damage-associated molecular patterns, normally cytoplasmic DNA, from disrupted cells and tissues. It has been shown previously that neutrophils are recruited to sites of sterile skin injury in a leukotriene LTB4 dependent process and occurs in very short time scales, thus we believe it is completely independent of the Notch-mediated recruitment of ILC3s and other innate immune cells^52^,^53^.

Recruitment of NKp46^low/−^ ILC3s into wounded dermis from the circulating blood was demonstrated by localization of dermal ILC3s after transfer of Rag2^−/−^ splenocytes into RORγ-deficient mice. ILC3s can be detected in human blood and in psoriasis lesions; the number of ILC3s in blood increased correspondingly with the influx of dermal ILC3s^23^. Recruitment of ILCs into mucosal epithelia is a CCL20/CCR6 dependent process^54^, and we hypothesized that Notch1-mediated, wound-induced ILC3 recruitment would employ similar mechanisms. However, results from genetic deletion of the CCL20/CCR6 or CXCL13/CXCR5 signalling in CCR6^−/−^ and CXCL13^−/−^ deficient mice suggest redundancy in cell recruitment mechanisms, implicating a potential role for other chemotactic factors including CXCL16, sphingosphine-1-phosphate, prosteoglandins, retinoic acid and other chemokines all known to be expressed in the skin^55^. CXCL12 may also function as a chemotactic factor. Although we detected no change in CXCL12 gene transcription within 24 h of injury (Fig. 5c), activated platelets are a potent source of CXCL12 (ref. 56). Thus, it cannot be ruled out that platelet-derived CXCL12 protein may contribute to ILC3 recruitment into skin wounds. Modulating TNFα levels had a direct consequence on the number of ILC3s at wound sites and the rate of wound healing. Moreover, Notch is a powerful inducer of TNFα and blocking TNFα activity downstream of activated Notch reduced the number of dermal ILC3s^9^.

RORγ^+^ ILC3s help facilitate skin repair through promoting epidermal re-epithelialization and monocyte/macrophage recruitment into the dermis in addition to the role of RORγ^+^ LTi cells in lymphoid tissue formation. Using a combination of Thy1.2 depleted Rag2^−/−^ mice and cell transfers we have shown there is no connection between lymphoid tissue formation and ILC3 function in skin wound repair. What remains uncertain is whether ILCs have discrete roles in both these processes, or whether detected changes in epithelial proliferation are a direct consequence of delayed early macrophage influx into wounds. ILC3s secrete CCL3, an important monocyte/macrophage chemokine attractant^57^, early (2 dpw) but not later (5 dpw) in the wound repair programme. RORγ^−/−^ mice had reduced levels of CCL3 mRNA at 2 dpw compared to wildtype controls and T lymphocyte deficient Rag2^−/−^ mice. These data suggest simplistically that, in the absence of ILC3s, reduced CCL3 production is the cause of delayed macrophage entry. However, in reality macrophage recruitment into injured tissues is much more complex. Macrophages themselves can produce CCL3 and other chemokines, including CXCL2 (also known as MIP2) and CCL2, have key recruitment functions^58^,^59^,^60^. Further, more recent work has identified that monocytes with distinct phenotypic characteristics are recruited into wound tissues at different times, suggesting an additional specificity of macrophage recruitment signalling that is yet to be fully understood^61^.

In conclusion, we propose that Notch1 acts a damage “sensor” in epithelial tissues, transmitting a “stress/injury signal” and instigating tissue repair through recruitment of ILC3s and macrophages. Given the importance of ILC3s and Notch signalling in maintenance and organization of the intestinal epithelium, we propose that it is likely a Notch-mediated “signal” is conserved amongst epithelial tissues required to undergo periodic repair.

## Methods

### Animal models

K14NICDER (C57BL/6 × CBA background) and RORγ^−/−^, Rag2^−/−^, CCR6^−/−^ and CXCL13^−/−^ mice in C57BL/6 background have been described previously^9^,^62^,^63^,^64^. All experimental procedures were performed with ethical permission by Durham and University of York under UK government Home Office licences to CA and MC (60/3941 and 60/4178). Experiments followed the national and institutional guidelines for the care and use of animals on the basis of the Animal (Scientific Procedures) Act 1986 and where possible ARRIVE guidelines. The effect of RORγ deletion on wound healing was previously uninvestigated, thus pre-specified effects prior to experimentation could not be determined and power calculations were not employed. Mice were housed in pathogen-free facilities and had access to food and water *ad libitum*. In K14NICDER mice, ER-inducible transgenes were activated by topical application of 2 mg of 4OHT (H6278, Sigma) dissolved in acetone.

For wounding experiments, mice were anesthetized using 2% inhaled isoflurane and then injected subcutaneously with the analgesic, Vetergesic (0.05 mg kg^−1^, Alstoe Animal Health). The back skin was shaved and sterilized with Videne surgical scrub (Ecolab) then rinsed with sterile water. Then two full-thickness, 4 mm diameter back skin wounds with a minimum distance of 1 cm apart were made using a punch biopsy (Stiefel). Some wounds were topically treated daily with recombinant TNFα (8 μg kg^−1^, product code: PNRMTNFAI, Thermo Scientific Pierce), TNFα blocking antibody (10 mg kg^−1^, Adalimumab, Humira, Abbott Laboratories) or PBS until humanely sacrificed 2 or 5 dpw^49^. Some mice were injected intraperitoneally with anti-NRR1 (5 mg kg^−1^, Genentech), anti-NRR2 (5 mg kg^−1^, Genentech) or control (anti-ragweed, Genentech) IgG (5 mg kg^−1^) for 7 days before wounding and then every 2–3 dpw for the duration of the experiment^30^. Some RORγ^−/−^ mice were injected intravenously with 1 × 10^6^ splenocytes derived from Rag2^−/−^ mice 24 h before wounding. Some Rag2^−/−^ mice were injected intraperitoneally with 200 ng anti-Thy1.2 antibody (BioXcell, 30H12) every other day for 2 weeks before wounding^24^,^44^. Mice were kept up to 8 dpw and wounds were measured with a calliper or quantified using a 3D camera (Quantificare).

Wounds were photographed using a 3D camera (Quantificare) during the healing response. Each wound was photographed three times at each time point, with each image analyzed three times to check for measurement consistency. Each image was assigned a random code and blind analysis was carried out using Dermapix software (Quantificare). Wound perimeters and diameters were recorded along with observations of the image (for example, excessive glare from the camera flash), and outliers excluded as appropriate when images could not be processed due to camera angle, excessive glare or obscured wound region.

In every experiment mice were age and sex matched, with wildtype littermate controls used when possible, and within each experiment 3 or more mice per genotype/experimental group were included. Experiments were repeated two to four times to confirm results. The majority of mice reported were females between 6 and 9 weeks of age. Occasionally, male mice or mice between 9 and 18 weeks were included in an experiment, however RNA and protein analysis was performed on age and sex-matched tissues and analysis was performed among experimental replicates where appropriate. Unexpectedly, rare RORγ^−/−^ mice developed leukaemia while undergoing experimental procedures and these mice were excluded from analysis. In addition, some larger wounds were made by 6 mm punch biopsy (Supplementary Fig. 7f) for wound closure study.

### Antibody labelling

Tissue was collected and processed as previously described^9^. 8 μm-thick frozen or paraffin sections were fixed in 4 or 0.4% paraformaldehyde, blocked in 10% goat or donkey serum, 0.25% fish skin gelatin and 0.2% bovine serum albumin, and then stained with antibodies diluted in blocking solution. Tissue sections were stained with fluorescent secondary antibodies (1:1,000, Invitrogen) and DAPI counterstained before imaging using a Leica Tandem SP5, Zeiss 710 or Zeiss 880 confocal microscope. For intracellular staining, frozen sections were fixed with 4% PFA, blocked as described above and incubated with fluorescence conjugated conjugated RORγ antibody diluted in permeabilization buffer (eBiosciences, cat. 00-8333) for 2 h. Brightness of images was adjusted using Adobe Photoshop CS3 software. See below for list of antibodies, suppliers and working dilutions.

### Flow cytometry

An 8 × 8 mm piece of mouse back skin including wounded area was removed from and the ventral surface was scraped gently with a scalpel to remove subcutaneous fat and muscle. Tissue was chopped into pieces and incubated with 0.35 mg ml^–1^ Liberase TL (Roche, Cat: 5401020001), 3 mg ml^−1^ Collagenase D (Roche, Cat: 11088866001) and 0.1 mg ml^−1^ DNase I (Roche, Cat: 10104159001) in RPMI Media 1640 (Life Technologies, Cat: 31870-025) at 37 °C for 2 h. After incubation, the digested tissue was put through a 70 μm strainer to isolate single cells. Cells were pelleted and re-suspended in 0.5% BSA/2 mM EDTA in PBS, then were blocked with anti-mouse CD16/32 (Biolegend, Cat:101320) and rat IgG (Sigma, Cat: I8015-10 mg) for 20 min before antibody staining for flow cytometry. For intracellular staining of RORγt, cells were first stained for surface markers as described above, then fixed overnight in Foxp3 Fixation/Permeabilization (eBiosciences Cat: 88-8824). After incubation, a RORγt antibody (BD, Q31-378, 1:100) was added to the cells for 1 h before analyzing cells.

For cytokine and chemokine staining, single cell suspensions were incubated with 50 ng ml^−1^ PMA (Phorbol 12-myristate 13-acetate; Sigma, P1585) and 5 ng ml^−1^ ionomycin (Sigma, 10634) in the presence of 10 μg ml^−1^ brefeldin A (Sigma, B5936) for 3 h before surface staining as described above. Cells were fixed in Foxp3 Fixation/Permeablization buffer (eBioscience, 88-8824) overnight, then antibodies to IL22, IL17A, IL17F and/or MIP1α (CCL3) and isotype controls were added to the cells for 1 h before analyzing cells. Cells were analyzed using a BD LSR Fortessa. See below for list of antibodies, suppliers and working dilutions.

### Antibodies for mouse flow cytometry and immunohistochemistry

*Flow cytometry*. CD45 (Biolegend, 30-F11, 1:100), Lineage cocktail (eBioscience, including17A2, RA3-6B2, M1/70, TER-119, RB6-8C5, 1:100), CD3 (eBioscience, 17A2, 1:100), CD3e (BD Biosciences, 145-2C11, 1:100), CD4 (eBioscience,RM4-5, 1:100), CD8 (Biolegend, 53-6.7, 1:100), F4/80 (eBioscience, BM8,1:400), CD11b (Biolegend, M1/70, 1:1000), Gr-1 (eBiosiences, RB6-8C5, 1:100), NK1.1 (Biolegend, PK136), NKp46 (Biolegend, 29A1.4, 1:100), CD117 (eBioscience, 2B8, 1:100), CD127 (eBioscience, A7R34, 1:100), CXCR5 (Biolegend, L138D7, 1:100), CXCR4 (Biolegend, L276F12, 1:100), CCR6 (Biolegend, 29-2L17, 1:100), ST2 (eBioscience, RMST2-2, 1:100), CD25 (Biolegend, PC61.5, 1:100), RORγt (BD, Q31-378, 1:100), IL22 (eBioscience, 1H8PWSR, 1:100), IL17A (Biolegend, TC11-18H10.1, 1:100), IL17F (Biolegend, 9D3.1C8, 1:100) and MIP1α (CCL3) (eBioscience, DNT3CC, 1:100).

*Immunohistochemistry*. F4/80 (eBioscience, BM8,1:400), CD3 (eBioscience, 17A2, 1:100), CD4 (eBioscience, RM4-5, 1:100), CD117 (eBioscience, 2B8, 1:100), CD127 (eBioscience, A7R34, 1:100), RORγt (eBioscience, AFKJS-9, 1:100 or BD, Q31-378, 1:100), activated Notch1 (Abcam, ab8925, 1:200), Ki67 (Abcam or Novocastra, NCL-Ki67p, 1:400), Gr-1 (eBiosiences, clone BM8, 1:100), α6 integrin (Abcam, 105669, 1:400). Jagged 1 (Santa Cruz Biotechnology, SC-6011, 1:100) and K10 (Biolegend, 905401, 1:1000).

### Western blotting

A small region of the skin containing the wound and the region just adjacent to the wound (approximately 0.5–1 cm^2^) was excised from the back skin. The tissue was homogenized using a polytron tissue homogenizer in RIPA lysis buffer (150 mM NaCl, 50 mM Tris–HCl (pH 7.5), 1% Nonidet P-40, 0.25% sodium deoxycholate with protease inhibitors). Lysate supernatants were run on a 6 or 10% polyacrylamide gel, transferred to PVDF membrane, blocked with 3% cold water fish skin gelatin (Sigma)/0.2% Tween-20/PBS and hybridized with antibodies to activated Notch1 (ab8925, 1:400, Abcam), activated Notch2 (ab8926, 1:400, Abcam) or beta-actin (A5441, 1:3000, Sigma). Blots were rinsed in 0.2% Tween-20/PBS, incubated with HRP-conjugated anti-rabbit or anti-mouse secondary antibody (Sigma) and visualized with ECL Western Blotting Substrate (Pierce). Following film detection of blots, band intensity was quantified using ImageJ and the mean band intensity value was calculated against the loading control (Fig. 1 and Supplementary Fig. 5). Images have been cropped for presentation (Supplementary Fig. 5). Full-size images of western blots are presented in Supplementary Fig. 9.

### RNA extraction and quantitative polymerase chain reaction

Wounded back skin tissues were collected as above and RNA was extracted from freshly-isolated tissues, from tissues snap frozen in liquid nitrogen at the time of collection or from tissues immersed in RNAlater (Invitrogen, R0901) for 24 h before freezing. Tissues were homogenized using polytron tissue homogenizer then RNA was isolated using RNeasy Mini Kit (Qiagen, 74104) following manufacturer's instructions including the optional, on-column DNAse I digestion step using RNase-free DNase set (Qiagen, 79254). An additional Proteinase K digestion step was performed in the protocol: homogenized lysates were incubated with 0.2 mg ml^−1^ Proteinase K (Sigma, P2308) in RLT buffer (Qiagen) at 55 °C for 10 min. Harvested RNAs were quantified using a NanoDrop microspectrophotometer and cDNA were prepared using High Capacity cDNA Transcription kit (Life Technologies, Cat: 4368814).

qPCR was performed using a StepOnePlus Real-Time PCR System instrument (Life Technologies, Cat. 4376600), with a standard quantitation-comparative Ct procedure as set by the manufacturer. Triplicate reactions (25 μl) of each experimental sample were prepared using CYBR master mix (Life Technologies, Cat: 4367659) and unlabelled primers for CXCL12, CXCL13, CCL3 and HPRT (control). Reactions were subjected to an initial 10-minute denaturation step at 95 °C, followed by 40 cycles of 95 °C for 15 s and 60 °C for 60 s. CCL20 and TNFα mRNA levels were examined using FAM probes and TaqMan Fast Master Mix (Life Technologies, 4352042) with a fast quantitation-comparative Ct procedure. Reference gene GAPDH was used as internal control. Reactions (20 μl) were subjected to an initial 20-s denaturation step at 95 °C, followed by 40 cycles of 95 °C for 3 s, 60 °C for 30 s.

An alternative qPCR method to quantify CCL20 (Supplementary Fig. 6f) was performed using a Rotor-Gene Q instrument (Qiagen), with a two-step rapid-cycling procedure as described by the manufacturer (Rotor-Gene Probe Handbook, Qiagen). Triplicate reactions (20 μl) of each experimental sample were analysed using a FAM probe for CCL20 and TaqMan Fast Universal PCR master mix (ABI). Reactions were subjected to an initial 3-minute denaturation step at 95 °C, followed by 45 cycles of 95 °C for 3 s and 60 °C for 10 s. Data were analysed using the Comparative Quantitation algorithm in the Rotor-Gene software, with calibrator samples for each run being compared in a common experiment. PCR reactions: 95 °C for 3 mins, followed by 45 cycles of 95 °C for 20s. A FAM probe for reference gene GAPDH was used to quantify genes of interest.

### Unlabelled primers and FAM probes for qPCR

All primers were used at a final concentration of 400 nM.

CXCL12: Forward 5′- CAGAGCCAACGTCAAGCA -3′, Reverse 5′- AGGTACTCTTGGATCCAC -3′

CXCL13: Forward 5′- CATAGATCGGATTCAAGTTACGCC -3′, Reverse 5′- TCTTGGTCCAGATCACAACTTCA -3′

CCL3: Forward 5′- GTTCTTCTCTGTACCATGAC -3′; Reverse 5′- CTCTTAGTCAGGAAAATGAC -3′

Control HPRT: Forward 5′- AGGAGTCCTGTTGATGTTGCCAG -3′, Reverse 5′- GGGACGCAGCAACTGACATTTCT -3′

Jagged1 Forward: 5′- AGAAGTCAGAGTTCAGAGGCGTCC -3′, Reverse: 5′- AGTAGAAGGCTGTCACCAAGCAAC -3′

Jagged2 Forward: 5′- CAATGACACCACTCCAGATGAG -3′, Reverse: 5′- GGCCAAAGAAGTCGTTGCG -3′

*FAM probes*. IL22: Mm01226722_g1, Life Technologies

CCL20: Mm01268754_m1, Life Technologies

TNFα: Mm00443258_m1, Life Technologies

GAPDH: Mm99999915_g1, Life Technologies

### Systems biology analysis of gene expression data sets

To determine key signalling pathways involved in Notch-mediated wound healing we used unbiased analysis of gene arrays from RORγ^+^ innate lymphoid cells (NIH GEO: GSE29777)^31^ and from uninjured, K14NICDER transgenic mouse back skin epidermis and dermis (NIH GEO: GSE23782)^9^. The MAS5-processed datasets of differentially expressed genes were imported into Ingenuity Pathway Analysis software (Ingenuity Systems, www.ingenuity.com) for analysis. All secreted, skin-derived factors that could interact with a receptor on ILC3s were determined. The highly upregulated genes in the epidermis and the dermis were filtered by the ‘extracellular matrix' gene ontology term. Ingenuity Pathway Analysis was used to build direct connections from the upregulated genes to their possible target receptors. The genes displayed in Fig. 5 were further selected to show the most probable/relevant/interesting interactions between secreted factors and their target receptors.

### Human cutaneous wound biopsies

Full-thickness punch biopsies (diameter 5 mm) were taken from the medial aspect of the upper arm of five healthy ethically consented Caucasian volunteers (male and female; aged 18–28; REC ethics reference: 09/H1012/3). These were left to heal by secondary intention. After 3 and 7 days the initial wound sites were biopsied again (diameter 7 mm) so that the entire wound site was collected with some adjacent uninjured wound margin tissue. Samples were snap frozen in liquid nitrogen immediately following biopsy, embedded in OCT (CellPath, UK) and set on dry ice. Samples were stored at –80 °C until cryosectioning for further analysis.

Cryosections of the tissue (6 μm) were allowed to reach room temperature and fixed in ice cold acetone at 4 °C for immunohistochemistry (IHC) or in formalin for H&E staining. Slides were then allowed to air dry at room temperature and washed in TBS before commencement of the immunohistochemical or histochemical protocol. For H&E, slides were first submerged in modified Harris Haematoxylin (Thermo Scientific, UK) for 4 min, washed in tap water and counterstained in Eosin (Thermo Scientific, UK) for 1 min. Stained slides were dehydrated through an ethanol gradient, cleared with xylene and mounted.

For IHC, a sequential triple stain was performed. Slides were blocked in TBS with 10% (v/v) normal donkey serum and normal human serum. After three washes with TBS, a first incubation with mouse monoclonal Anti-RORC (1:50, MABF81, Millipore) and rabbit monoclonal anti-CD3 (1:100, AC-004 A, Epitomics) was performed for 1 h at room temperature. Sections were washed with TBS-tween-20 (0.01% (v/v) then incubated with secondary antibodies (Alexa-488 Donkey anti-mouse, 1:250; Alexa-555 Donkey anti-rabbit, 1:150) for 1 h at room temperature. After washing again, sections were blocked with TBS containing 10% (v/v) normal mouse serum. A second incubation with the third primary antibody, mouse monoclonal anti-CD127 (IL-7Ra) (1:100, A18684, Life Technologies), was then performed for 1 h at room temperature, followed by washing with TBS-Tween-20. Sections were then incubated with alexa-647 donkey anti-mouse (1:250) for 1 h at room temperature, washed and counterstained with DAPI (1:500) for 15 min at room temperature. Slides were given a final wash, dried and mounted using Vector Shield. Slides were scanned on a Pannoramic 250 Flash II, 3DHistech Ltd. and analysed through the Pannoramic Viewer platform (v.1.15.2 created by 3DHistech Ltd).

### Primary dermal cells

Primary mouse fibroblasts were isolated using a method on the basis of Jahoda *et al*.^65^. In brief, after sacrificing the mouse, the back skin hair was clipped and then the skin tissue and washed in DMEM (Invitrogen) with double strength antibiotics (2 × Pen/Strep—Penicillin 100 U ml^−1^ Streptomycin 100 μg ml^−1^). The fat and non-dermal tissue was gently scraped from the tissue before cutting into pieces of ∼10 mm^2^. Each piece of tissue was cut repeatedly using curved blade scissors until it became slurry and the tissue was spread onto the bottom of a 6-well plate. The tissue was then covered with DMEM+10% foetal calf serum and 1 × Pen/Strep (Fisher) and placed in a 37 °C incubator at 5% CO_2_. After 12 days cells were passaged.

P1 mouse fibroblasts 90% confluent were treated with either 1:1,000 TNFα (20 ng ml^−1^, product code: PNRMTNFAI, Thermo Scientific Pierce), 1:1,000 TNFα blocking antibody (50 μg ml^−1^, Adalimumab, Humira, Abbott Laboratories) or both diluted in DMEM+10% FCS (PAA Laboratories) and 1 × Pen/Strep. Some cells were left untreated. Cells were incubated for an additional 24 h then lysed and RNA collected using an RNAeasy Mini Kit (Qiagen) following the manufacturer's protocol.

### Statistics

For western blotting and mRNA quantification, samples were normalized where the average of the uninjured skin control, unless otherwise stated, was designated as 1 or 100%. The number of independent samples is stated in the figure legends. Statistical analysis was performed using SPSS (IBM) and Prism GraphPad software. Data variances were calculated and normal distribution of the data was determined by Kolmogorov–Smirnov tests. Normally-distributed data were analysed by two-way ANOVA with statistical significance between zones at calculated by Bonferroni's multiple comparison test or Student's *t*-test. Non-normally-distributed data was analysed using a Mann–Whitney test. % wound openness (Fig. 7b and Supplementary Fig. 7) was calculated using a linear mixed effects model. Specific *P*-values are included on figures or in figure legends.

## Additional information

**How to cite this article:** Li, Z. *et al*. Epidermal Notch1 recruits RORγ^+^ group 3 innate lymphoid cells to orchestrate normal skin repair. *Nat. Commun.* 7:11394 doi: 10.1038/ncomms11394 (2016).

## Supplementary Material

Supplementary Information Supplementary Figures 1-9
