# Supplementary material for: Epidermal Notch1 recruits RORγ+ group 3 innate lymphoid cells to orchestrate normal skin repair
Source: Nat Commun. 2016 Apr 21;7:11394. doi: 10.1038/ncomms11394 (PMC4844683; doi:10.1038/ncomms11394)
Supplement: Supplementary Information — Supplementary Figures 1-9 [file ncomms11394-s1.pdf]

Supplementary Figure 1:

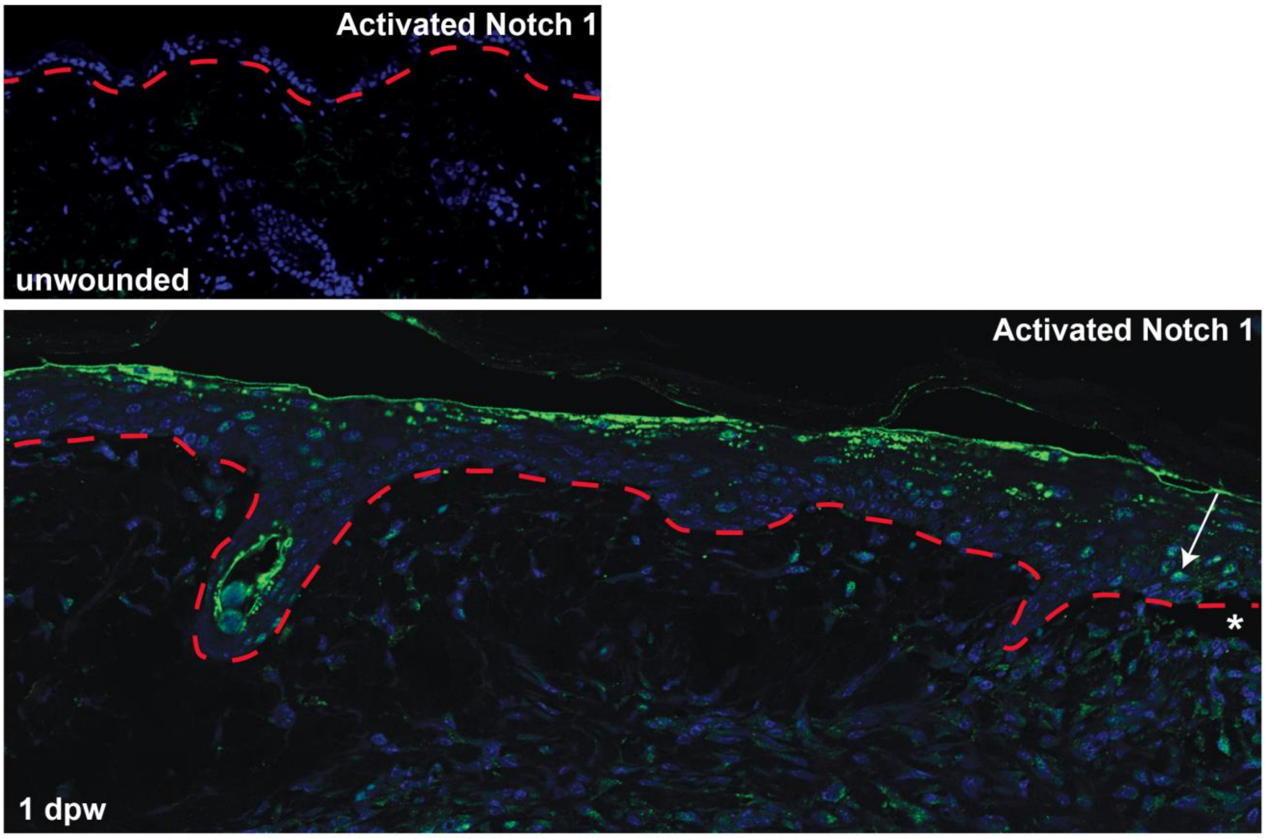

Supplementary Figure 1: Notch1 is activated in the epidermis by wounding.

Tissue sections of unwounded skin or wounded skin 1 days post wounding (dpw) stained with antibodies to cleaved, activated Notch1 (green) and counterstained with DAPI (blue). White arrow indicates a representative example of an antibody-stained cell. Note activated Notch1 is detected mostly in the suprabasal cells. Red, dashed line marks epidermal/dermal boundary. Asterisk (lower panel) marks edge of wound.

Supplementary Figure 2:

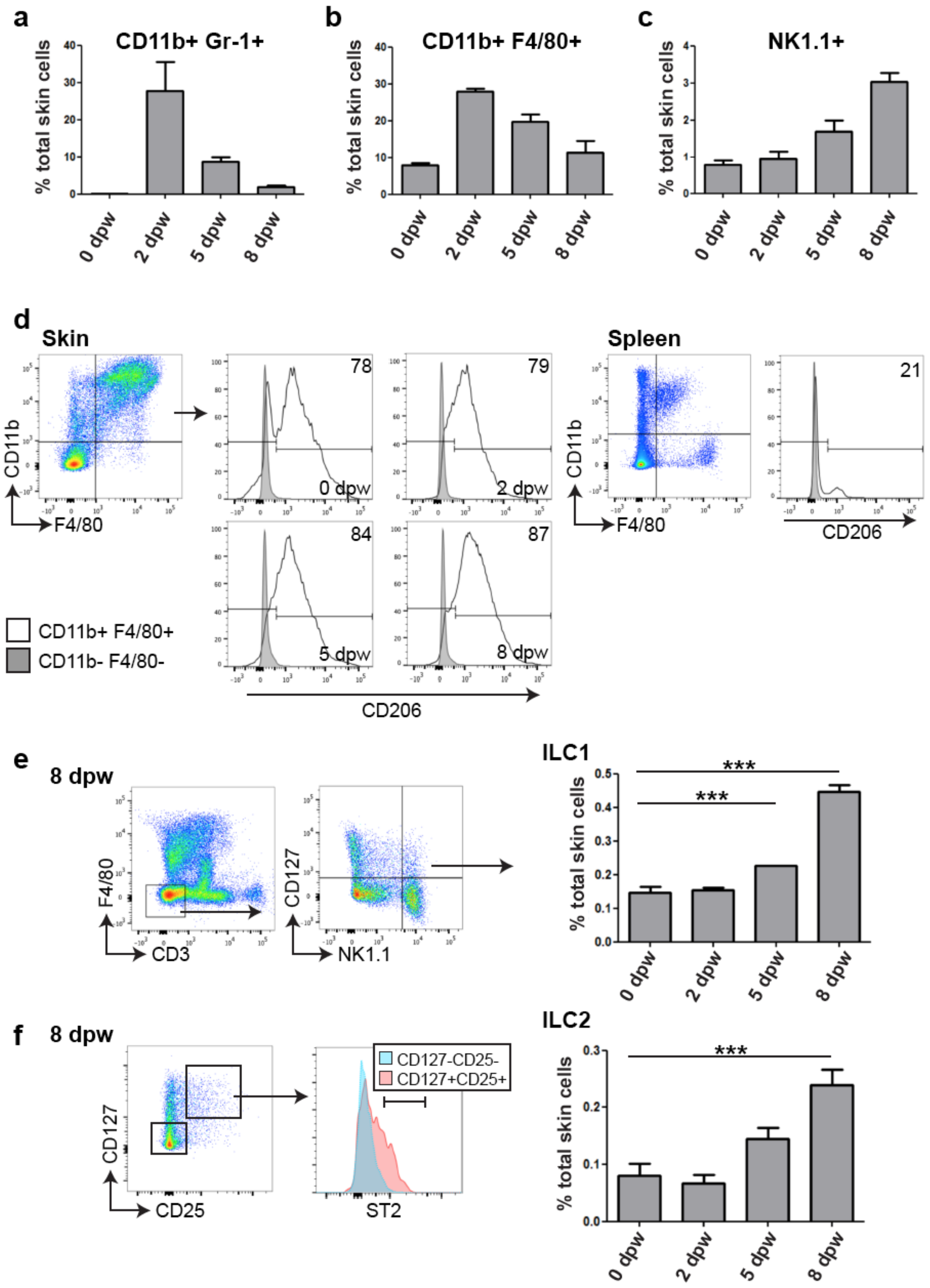

Supplementary Figure 2: Innate immune response following wound healing.

Leukocyte populations present in unwounded and wounded back skin 2, 5 and 8 dpw were quantified by flow cytometry (n=3 mice per group). Wounded and unwounded back skin was digested to a single cell suspension and cells were labelled with antibodies to CD45, CD3, CD11b, CD25, Gr-1, F4/80, NK1.1, CD206, CD127 and/or ST2. Labelled cells were gated first on CD45 and subsequently as shown in panels a-f. (a-c) Wounding causes early recruitment of $CD11b^{+}Gr\text{-}1^{+}$ neutrophils (a) and $CD11b^{+}F4/80^{+}$ monocyte/macrophages (b); the greatest percentage of NK cells ($NK1.1^{+}CD127^{-}$; panels c, e) was later in wound healing. In panel d, $CD11b^{+}F4/80^{+}$ macrophages were phenotyped by the expression of CD206 and numbers on histograms show percentage of $CD11b^{+}F4/80^{+}CD206^{+}$ cells at 0, 2, 5 and 8 dpw. Labelled isolated spleen cells were used as controls for CD206 staining in panel d. (e, f) To calculate the percentage of ILC1s, cells were initially gated for $CD45^{+}CD3^{-}F4/80^{-}$ expression; ILC2s were initially gated for $CD45^{+}Lin^{-}$ expression. ILC1s and ILC2s were subsequently quantified using the gating strategies as shown in panels e and f. Like ILC3s, these cell populations constitute a very small fraction of the total cells. Wounding induced recruitment of ILC1s and ILC2s, in the later stages of healing. A statistically significant increase in ILC1 numbers was detected at 5 and 8 dpw, and ILC2 numbers only at 8 dpw. Normally distributed data were analysed by two-way ANOVA with statistical significance between zones at calculated by Bonferroni's Multiple Comparison Test ($p \leq 0.05$; ***). Error bars represent standard error of the mean.

Supplementary Figure 3:

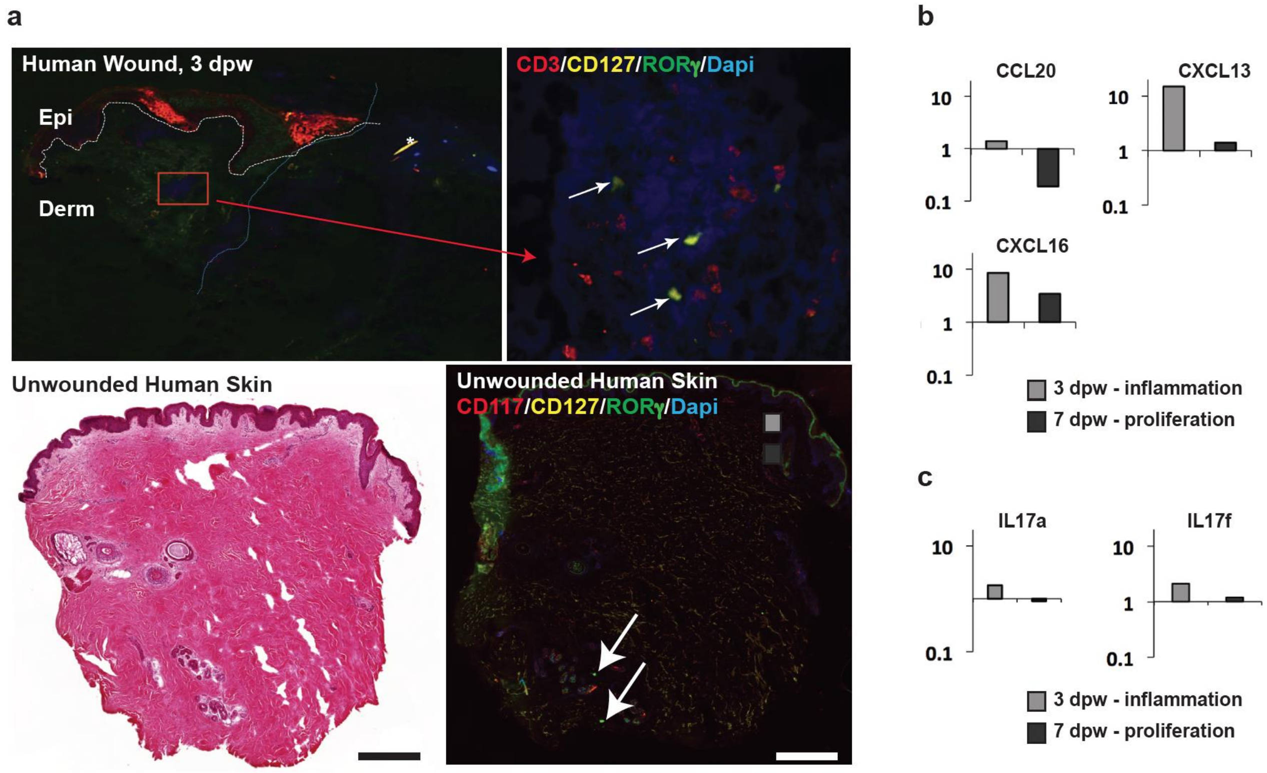

Supplementary Figure 3: Injury recruits ILC3 into human wounds.

(a) Sections from unwounded (bottom panels) or human wound 3 days post wounding reacted with antibodies to CD3 (upper right panel, red), CD117 (bottom right panel, red) CD127 (yellow) and RORγ (green) and counterstained with DAPI (blue). Red box denotes area of higher magnification in upper right panel. Epi = epidermis; Derm = dermis. Dashed line marks epidermal/dermal boundary; asterisk marks site of wound; white arrows mark RORγ^{+} cells. Scale bars equal 100 microns. (b, c) Relative mRNA levels of human chemokines and cytokines CCL20, CXCL13, CXCL16, IL17a and IL17f in human wounds 3 days post wounding during the inflammatory wound healing phase and 7 days post wounding during the proliferation phase. mRNAs were quantified by qRT-PCR and levels were normalised RNA levels in unwounded human skin (designated 1). Data plotted on a log scale; 3 biological replicates.

Supplementary Figure 4:

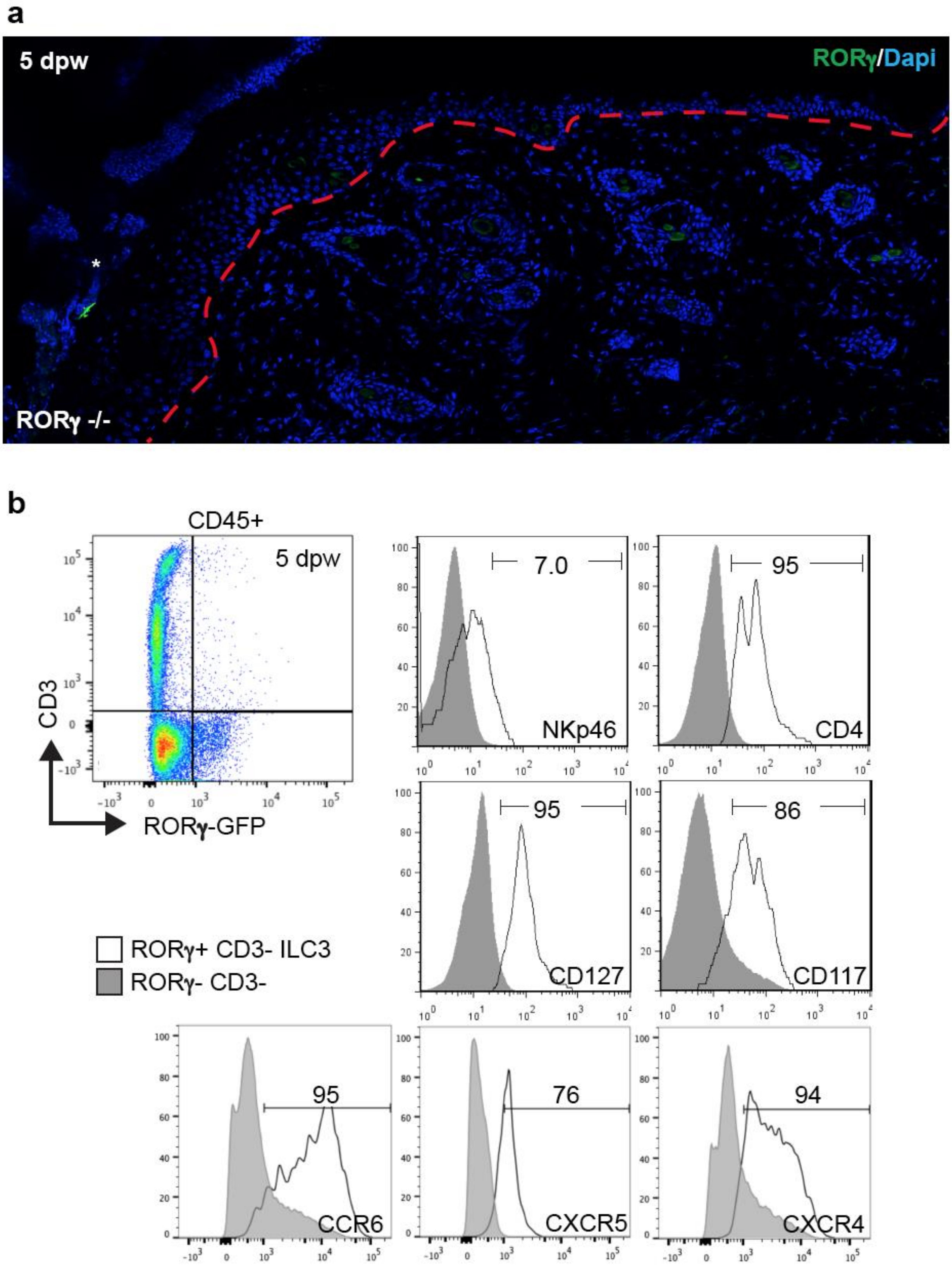

Supplementary Figure 4:

RORγ^+ ILC3s are recruited in the post-wounding inflammatory response. (a) Back skin sections from a punch-wounded (4 mm) ROR$\gamma^{-/-}$ mouse collected 5 days post wounding (dpw) stained with an antibody to RORγ (green) and DAPI counterstained (blue). Red, dashed line marks epidermal/dermal boundary. Asterisk marks edge of wound. (b) Dorsal back skin from ROR$\gamma^{eGFP/+}$ from punch-wounded (4 mm) animals 5 dpw. Single cell suspensions of whole back skin were co-stained with antibodies against CD45, CD3, CD117, CD127, NKp46, CCR6, CXCR4 and/or CXCR5 prior to analysis by flow cytometry. Cells were initially gated based on CD45 expression and subsequently gated as shown. Histograms show expression of the cell surface markers on gated $CD3^-$RORγ-$eGFP^+$ cells (white peak) and $CD3^-$RORγ-$eGFP^-$ (grey peak) populations. Numbers indicate percentage of $CD3^-$RORγ-$eGFP^+$ cells expressing respective markers.

Supplementary Figure 5:

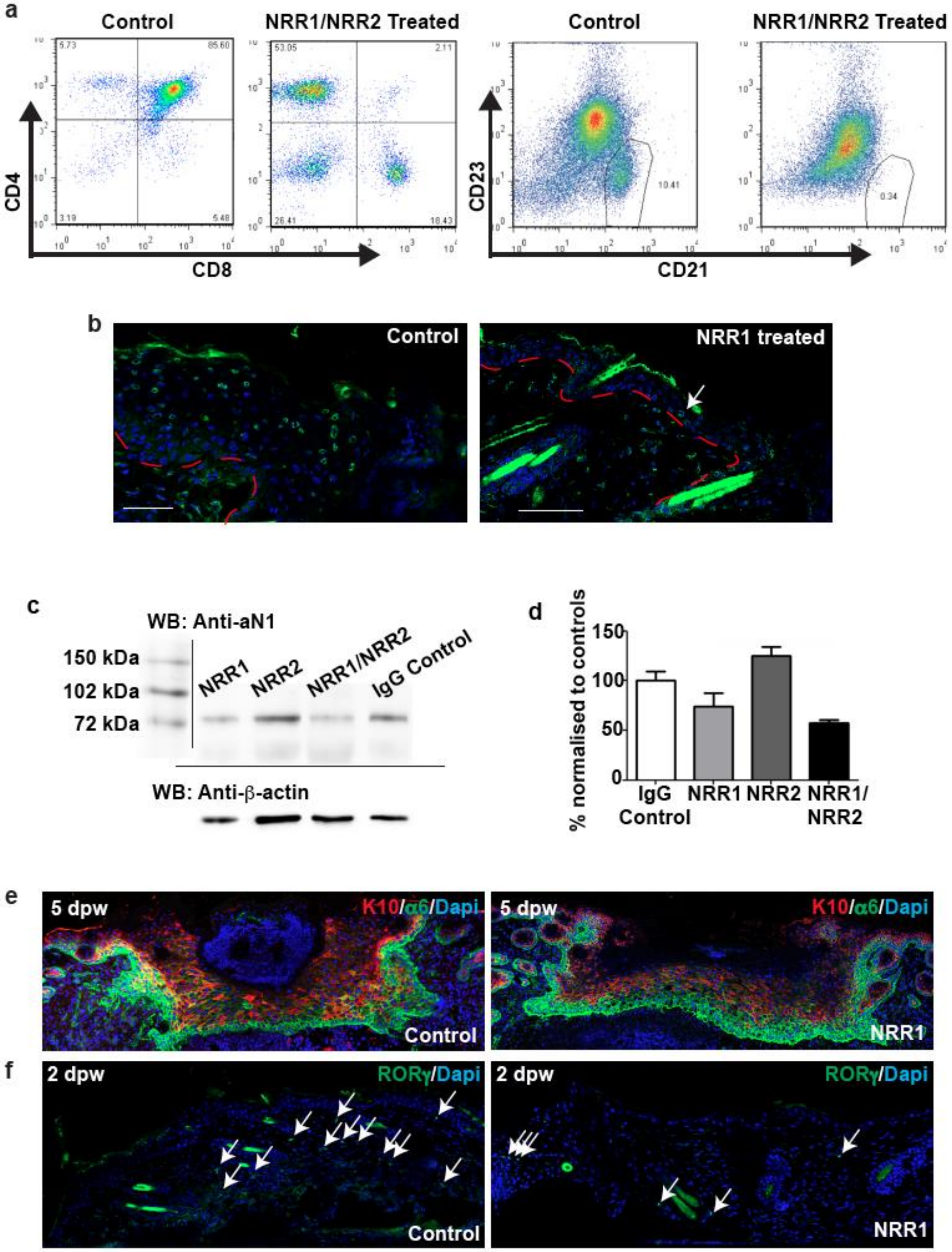

Supplementary Figure 5:

NRR1 and NRR2 antibodies block Notch1 receptor and Notch2 receptor function *in vivo*. (a) Cells were isolated from the spleen and thymus from mice injected with 5 mg/kg of NRR1 and NRR2 antibodies or 5 mg/kg IgG isotype control for 7 days. Cells were analysed by flow cytometry analysis. (a) Thymocytes were labelled with CD4 and CD8 antibodies, only 2.1% of cells are $CD4^{+}CD8^{+}$ in NRR1/NRR2 treated mice. Functional Notch1 signaling is required for T cell maturation. Spleen cells were labelled with B220, CD23 and CD21 antibodies. NRR1/NRR2 treatment leading to the loss (0.3%) $CD21^{hi}/CD23^{lo/-}$ marginal zone B cells. (b - e) Mice were injected with 5 mg/kg of NRR1, NRR2, both NRR1 and NRR2 antibodies or an IgG isotype control for 7 days prior to wounding. Tissues collected 2 or 5 dpw were processed for sectioned antibody staining or Western immunoblotting of protein lysates. (b,e,f) Wounds 2 (b,f) or 5 (e) days post wounding were sectioned and stained with antibodies to cleaved, active Notch1 (b, green), Keratin 10 (e, K10, red), alpha 6 integrin (e, α6, green) and/or RORγ (f, green) and DAPI counterstained (b, e, f, blue). Note in panel b, significant reduction in epidermal staining of activated Notch1 in NRR1 treated tissues; white arrow in b, right panel marks one cell in the epidermis. In panel f, white arrows mark $ROR\gamma^{+}$ cells in the dermis. (c,d) Western blotting of protein lysates were probed with antibodies to cleaved, active Notch1 (anti-aN1) or beta-actin as a loading control (See Supplementary Fig.9 for images of full blots). (d) Immunoblots were quantified using ImageJ and the mean band intensity value was calculated against the loading control. Graph bars in d represent mean of 3 biological replicates and error bars = standard error of the mean. Red, dashed line (b) marks epidermal/dermal boundary; scale bars equal 50 microns. kDa = kilodaltons. Experiments were repeated ≥ 2 times.

Supplementary Figure 6:

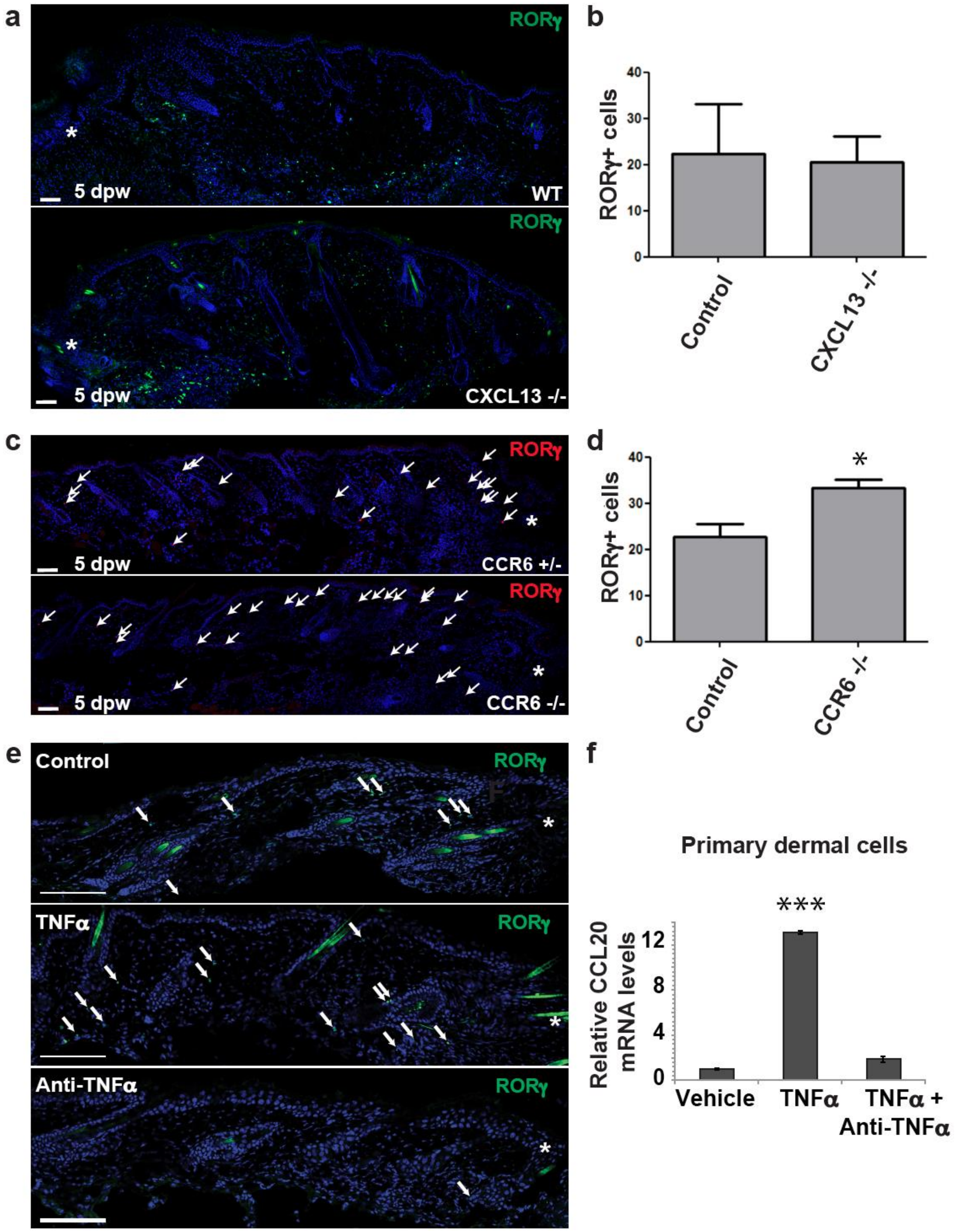

Supplementary Figure 6: CXCL13-/- and CCR6-/- mice have normal ILC3 recruitment into skin wounds; TNFα levels are important for RORγ^{+}ILC3s localisation to wounded sites

(a,c) Sections of wounded back skin 5 days post injury from CXCL13$^{-/-}$ (a) or CCR6-/- (c) mice and appropriate controls: wild type (WT) colony mates of CXCL13$^{-/-}$ mice (a) or CCR6$^{+/-}$ littermates (c) of CCR6 mutants. (a,c). Antibody labelling of RORγ (green, a; red, c) and DAPI counterstained (blue). (b,d) ILC numbers were quantified in 3 sections of 3 biological replicates; no difference in ILC recruitment was detected in CXCL13$^{-/-}$ wounds but a small, but statistically significant, increase in CCR6$^{-/-}$ wounds was detected ($p = 0.036$; Student's t test; *). (e) Wild type mice were punch wounded and treated with PBS (control), TNFα or TNFα blocking antibody (anti-TNFα) daily (n = 4 control; n = 7 TNFα antagonist; n = 8 TNFα). Sections of tissues collected 5pdw were stained with antibodies reactive to RORγ (e, green) and counterstained with DAPI. (f) Primary dermal cells treated *in vitro* with TNFα or TNFα and TNFα blocking antibody (Anti-TNFα) for 6 hours. CCL20 mRNA levels were quantified by qPCR; data were normalised to vehicle-only treated cells and significance determined by Student's t test ($p < 0.001$; 3 replicate experiments). Graph bars represent experimental mean of technical replicates (n = 3) and error bars represent standard error of the mean (s.e.m.); asterisks mark site of wound; scale bars equal 100 microns; white arrows mark ILCs (c,e); experiments were repeated ≥ 2 times.

Supplementary Figure 7:

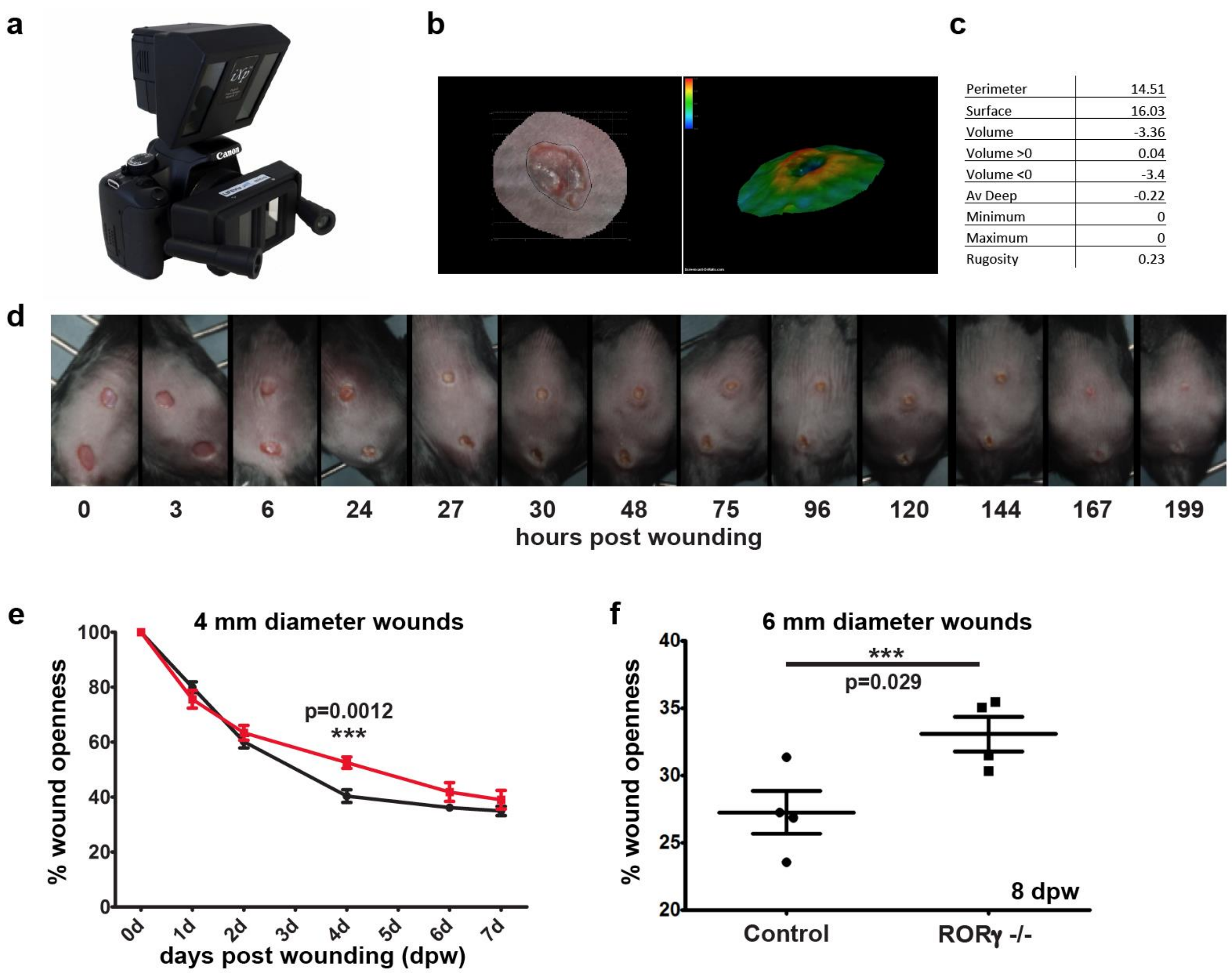

Supplementary Figure 7: 3D quantification of wound dimensions in live control and RORγ-/- animals.

(a-d) Wound closure was quantified in 3D for some animals. Using a 3D camera developed by Quantificare (a), 3D images including topographical measurements were captured of wounds (b, c). (c) An example of data measurements collected. (d) Non-invasive 3D image capture allowed the same animals to be imaged repeatedly. (e, f) Wound openness, as a percentage of initial wound size, calculated daily for up to 7 dpw by 3D quantification in wild type and $ROR\gamma^{-/-}$ mice (n ≥ 4 wounds per data point). (e) Graph of wound closure in small, 4 mm diameter wounds over time. Red line = $ROR\gamma^{-/-}$ wounds;

black like = wild type wounds. (f) Wound openness 8 dpw in mice receiving large, 6 mm wounds. Note wound closure is slower in larger wounds and wound closure defects were exacerbated in mice with larger wounds. Significance determined by comparing data for each day post wounding (Student's t test; p values included on graphs).

Supplementary Figure 8:

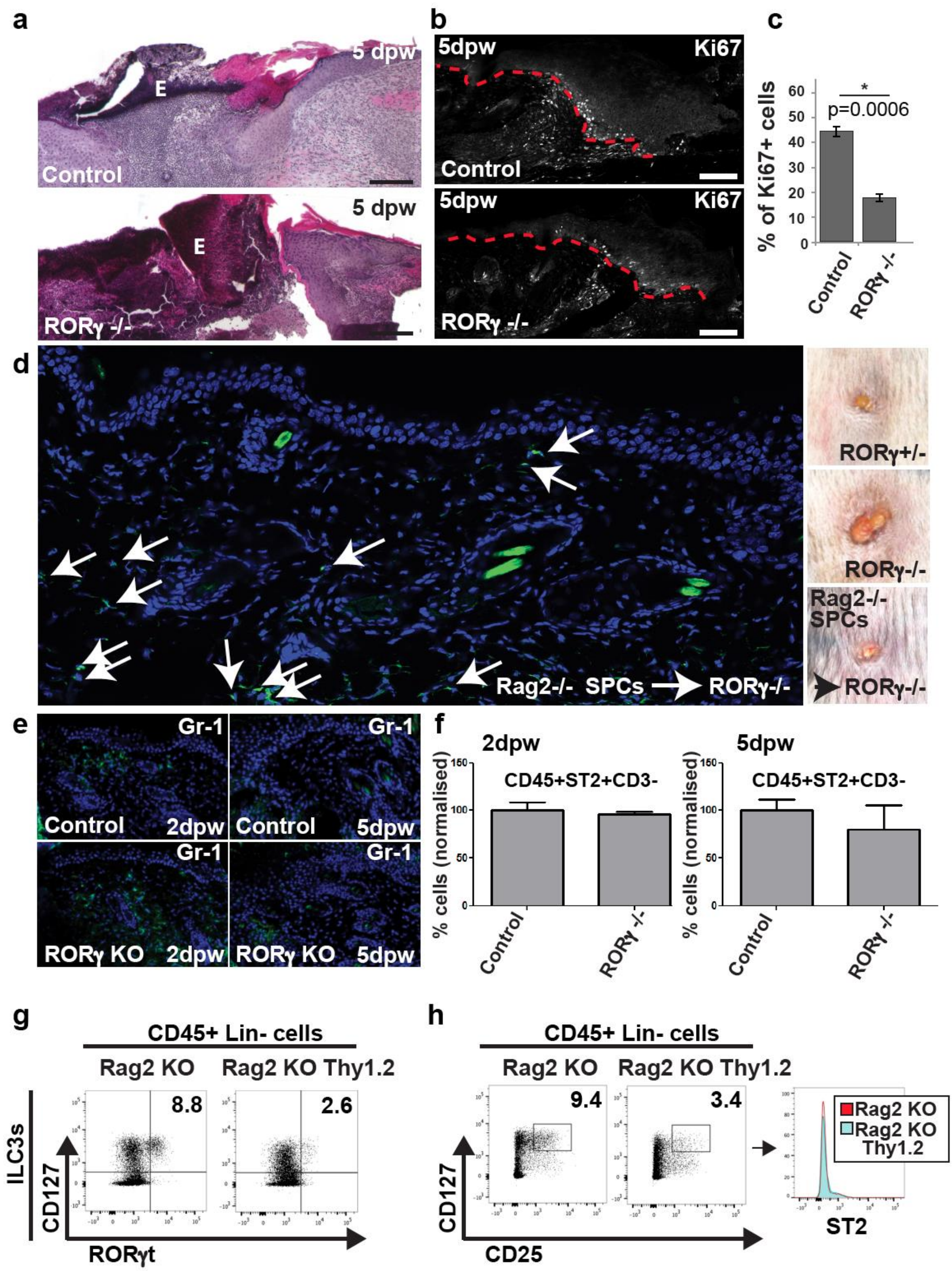

Supplementary Figure 8: Epidermal proliferation, but not neutrophil or ILC2 recruitment is delayed in wounded RORγ$^{-/-}$ back skin; transplantation of ILC3-containing splenocytes into RORγ$^{-/-}$ mice rescues wound closure delay

(a-c,e,f) 4 mm wounds were created in RORγ$^{+/-}$ (control) and RORγ$^{-/-}$ mice. Tissues were collected 2 or 5 dpw. (a) Hemotoxylin and eosin stained back skin sections of tissues 5 dpw. A thick, dense eschar (E) is evident in the RORγ$^{-/-}$ wounds. (b) Sections of wounded RORγ$^{-/-}$ and control tissues stained with antibodies to Ki67 (white). (c) Graph shows quantification in antibody stained sections (b) of Ki67^{+} basal epidermal cells within 500 microns of the wound site in control verses RORγ$^{-/-}$ mice at 5 dpw (Student t test; $p = 0.0006$). Experiment repeated ≥ 3 times. (d) Rag2$^{-/-}$ spleen cells (Rag2$^{-/-}$ SPCs) were transplanted into RORγ$^{-/-}$ mice prior to wounding (n = 3 mice). At 5 dpw wound size remained open in untransplanted RORγ$^{-/-}$ mice (middle image) while wounds of RORγ$^{+/-}$ mice (top image) and splenocyte-transplanted RORγ$^{-/-}$ mice (bottom image) were less open and of similar size to each other. Tissue sections antibody stained for RORγ□ILC3s were detected in wound sites in RORγ$^{-/-}$ skin with transplanted Rag2$^{-/-}$ spleen cells. (e) Sections of RORγ$^{+/-}$ (control) and RORγ$^{-/-}$ (RORγ KO) wounded tissues stained with an antibody to Gr-1 antigen (green) and DAPI counterstained (blue). (f) Quantification by flow cytometry of CD45^{+}Lin^{-}ST2^{+}CD3^{-} ILC2s in control and RORγ$^{-/-}$ skin 2 and 5 days post wounding (n=3 per time point). Note no difference in ILC2 recruitment in wild type and mutant skin was detected. (g,h) Rag2$^{-/-}$ (Rag2 KO) mice were injected intraperitoneally with anti-Thy1.2 antibodies or saline every other day for 2 weeks prior to wounding. Skin wounds were isolated 2 dpw and the percentage of CD45^{+}Lin^{-}CD127^{+}RORγt^{+} cells (ILC3s) and CD45^{+}Lin^{-}CD127^{+}CD25^{+} cells was calculated by flow cytometry. Histograms (h) show a similar proportion ST2^{+} cells in gated CD45^{+}Lin^{-}CD127^{+}CD25^{+} population in both Thy1.2 treated and untreated Rag2$^{-/-}$ mice (n=3 per treatment). White arrows (d) mark RORγ^{+}

positive cells; graph error bars represent standard error of the mean (s.e.m.); scale bars equal 100 microns.

Supplementary Figure 9:

A

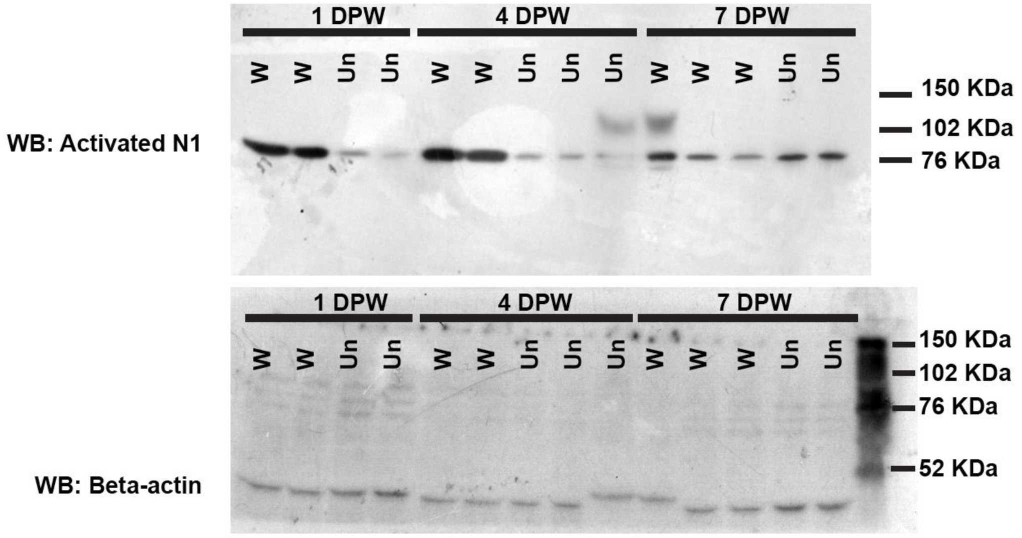

B

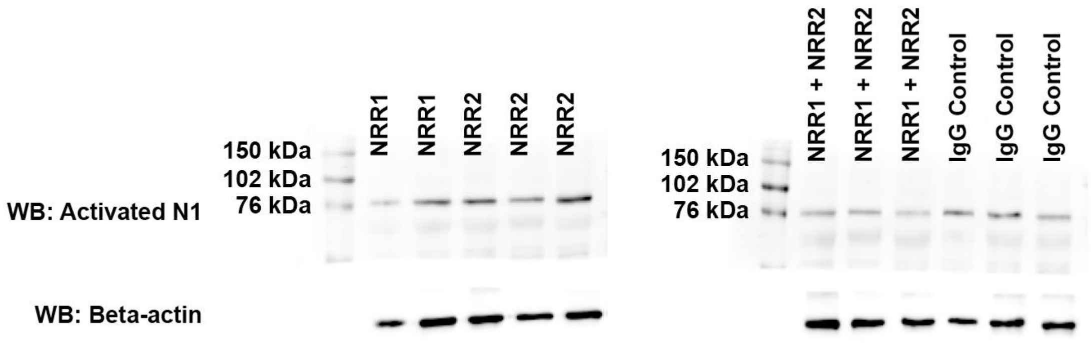

Supplementary Figure 9: Full-size images of Western blots. (a) Back skin tissues from punch-wounded wild type mice were collected 1, 4 and 7 days post wounding (DPW). Protein levels of cleaved, activated Notch1 proteins (Activated N1) in wounded skin (W) were quantified by antibody detection in a Western immunoblotting assay and β-actin protein (Beta-actin) detection was used to normalize protein loading. As an experimental control, unwounded back skin (Un) was taken from a punch-wounded mouse at a distal site (minimum 2 cm) from wound site. (b) Wild type mice were injected with 5 mg/kg of NRR1, NRR2, both NRR1 and NRR2 antibodies (NRR1+NRR2) or an IgG isotype control for 7 days prior to wounding. Tissues were collected 2 dpw and protein lysates were probed with antibodies to cleaved, active Notch1 or β-actin in a Western immunoblotting assay.
